# History and Science behind the Eating Assessment Tool-10 (Eat-10): Lessons Learned

**DOI:** 10.1007/s12603-023-1950-9

**Published:** 2023-07-28

**Authors:** A. Schindler, M. de Fátima Lago Alvite, William Gildardo Robles-Rodriguez, N. Barcons, P. Clavé

**Affiliations:** 1 Department of Biomedical and Clinical Sciences ‘Luigi Sacco', University of Milan, Milan, Italy; 2 Fonoaudiology, Placi Hospital, Rio de Janeiro, Brazil; 3 Facultad de Medicina, Fundación Universitaria de Ciencias de la Salud, Bogotá, Colombia; 4 Medical Affairs, Nestlé Health Science, Vevey, Switzerland; 5 Gastrointestinal Physiology Laboratory, Hospital de Mataró, Universitat Autónoma de Barcelona, Mataró, Spain; 6 Centro de Investigación Biomédica en Red de Enfermedades Hepáticas y Digestivas (Ciberehd), Instituto de Salud Carlos III, Barcelona, Spain

**Keywords:** Aged, deglutition disorders, early diagnosis, patient health questionnaire, preventive medicine

## Abstract

**Introduction:**

Oropharyngeal dysphagia (OD) is an underdiagnosed medical condition with a high prevalence in populations such as patients with frailty, neurological disease, or head and neck pathology. Potential barriers to its diagnosis include lack of (or low) awareness of the existence and severity of the condition, the hidden nature of the condition within the ‘normal ageing' process, clinical limitations, and socioeconomic reasons. Consequently, an effective treatment is not systematically offered in a timely manner, and complications, such as dehydration and respiratory infections or aspiration pneumonia, can arise. To overcome this issue, the early use of screening questionnaires to identify people at risk of swallowing disorders represents the cornerstone of preventive medicine. Several screening tools have been created but few are widely used in clinical practice. The Eating Assessment Tool-10 (EAT-10) was developed as a quick, easy-to-understand, and self-administered screening tool for OD.

**Methods:**

A literature review was conducted in five databases with no restrictions on the language, date of publication, or design of the study to identify aspects of the validation, applicability, and usefulness of EAT-10.

**Results and Conclusions:**

Transcultural adaptation and translation studies, as well as studies involving various types of patients with dysphagia in different settings have shown the validity and reliability of EAT-10 in relation to the gold standard and other validation tools. The use of this standardised screening tool could be used as a primary screening instrument of dysphagia in routine clinical practice across a wide range of diseases and settings and thereby increase the likelihood of early diagnosis and management of a condition that lead to serious complications and impaired quality of life.

**Figure:**
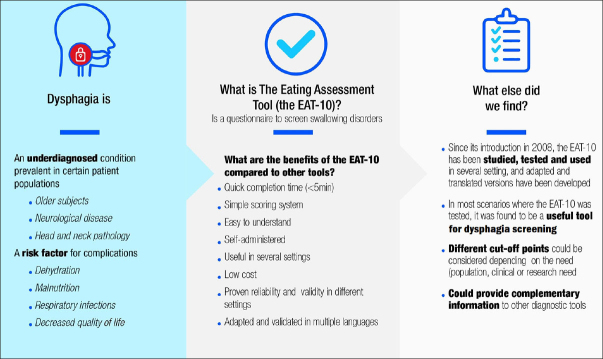

## Introduction

**Figure 1:**
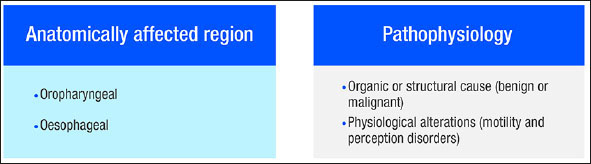
Dysphagia classification

**D**ysphagia is defined as difficulty in the formation or movement of the food bolus from the mouth to the stomach in a safe manner or, simply put, as any type of difficulty that occurs during swallowing (1, 2). Despite being considered a symptom, the World Health Organization has included dysphagia in the International Statistical Classification of Diseases (ICD) with code R13.10 (3), as well as in the International Classification of Functioning, Disability and Health (ICF) with code S330 (4). Dysphagia has been classified by the Canadian Association of Gastroenterology according to various characteristics, as shown in Figure 1 (1, 5).

Oropharyngeal dysphagia (OD) commonly presents with difficulty in the initial stages of swallowing and is usually due to structural, anatomical, or neuromuscular alterations. Oesophageal dysphagia occurs after swallowing and, in most cases, is the result of intrinsic structural conditions, extrinsic compression, and motility disruption (6). The populations with the highest risk of presenting OD are described below (1).

• Older adults
  Dysphagia occurs in 30%–40% of people over the age of 65 (7). It is estimated that it affects 10 to 33% of older adults and more than 16 million Americans live with this condition (8, 9); it is related to older age, male sex, having comorbidities (10) and it is mainly explained by anatomical, neural, and muscular changes characteristic of the ageing process. Minor modifications in the swallowing process due to age can be observed in healthy adults, which account for a decrease in the isometric tongue pressure (known as presbyphagia). While it rarely causes OD, it can potentially progress to dysphagia in the presence of other stress factors such as lifestyle or medications (11). OD has been recognised as a geriatric syndrome in a position document developed by the Dysphagia Working Group (12). OD matches the definition of a geriatric syndrome because it is highly prevalent among older people, is caused by multiple factors, is associated with several comorbidities and a poor prognosis, and needs a multidimensional approach to be treated On the other hand, certain medications (such as antidepressants, antipsychotics, or sedatives) have been correlated with OD in this population (13). Frailty and sarcopenia are also closely related to the pathophysiology of OD (14).
• Patients with neurological or neurodegenerative disease
  Around 64%–78% of patients with acute stroke exhibit OD, and in chronic phases of neurological disease, this number has been estimated at 40%–81% (15). Other diseases with a high prevalence of dysphagia include advanced Parkinson's disease (52%–82% of patients) and multiple sclerosis (30%–40%). In advanced stages of amyotrophic lateral sclerosis, dementia, and oculopharyngeal muscular dystrophy, 80%–100% of patients present with dysphagia (16, 17). In view of the high incidence of OD in patients with stroke, dysphagia assessment is considered in the Stroke Code, with the aim of reducing the severity and sequelae derived from such an event (18, 19).
• Patients with head or neck pathology
  Dysphagia occurs due to tumours in the head and neck and as a consequence of different types of treatment (20). The prevalence is up to 44% in patients who have received chemoradiotherapy, and close to 10% in patients who have undergone surgery. Other conditions that can lead to dysphagia include throat or larynx trauma, post-intubation and tracheostomy states, cervical surgery, and congenital malformations (21, 22).

Even with the simplistic approach of defining OD solely as difficulty swallowing, the particularities implicit in its etiological process are diverse and sometimes complex. Furthermore, patients may not always be aware that they have dysphagia, a common occurrence in patients with Parkinson's disease and other neurodegenerative diseases (23).

**Figure 2:**
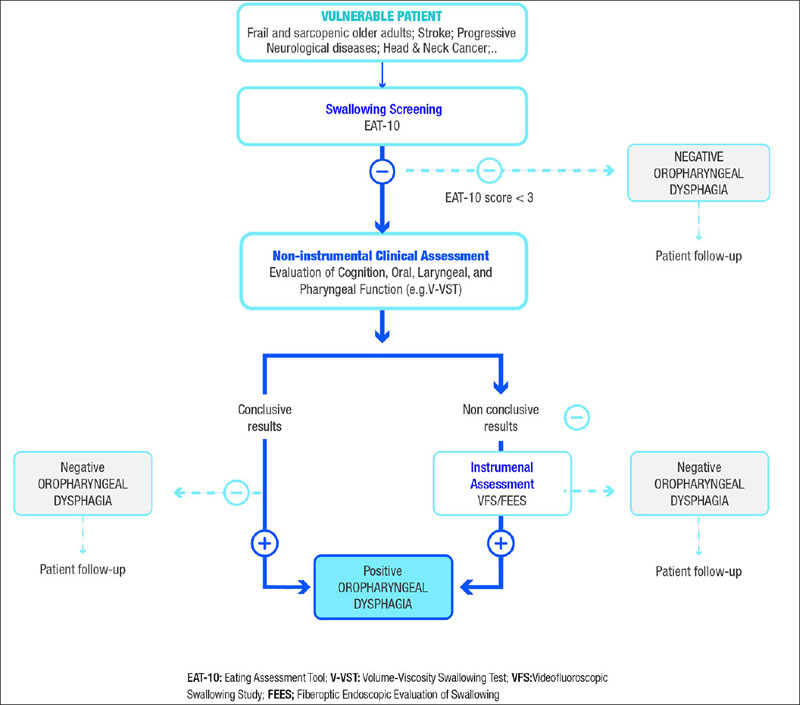
Screening and diagnostic algorithm of oropharyngeal dysphagia

Adapted from Cabré et al. (24)

The diagnostic algorithm of OD (Figure 2) requires a three-step approach consisting of clinical screening and clinical and instrumental assessments. Patients who have ‘failed' the screening test are at risk of OD and require further clinical and/or instrumental assessment(s). The screening phase aims to identify patients at risk for OD, the clinical assessment aims to identify clinical signs and symptoms for OD, and the instrumental assessment aims to identify the mechanisms and pathophysiology of impaired safety and/or efficacy of swallowing and to select the optimal treatment (24).

Although the first step in diagnosing dysphagia consists of obtaining a detailed patient history and conducting a physical examination, an accurate diagnosis is only obtained through the use of tests such as a videofluoroscopic swallow study, a barium oesophagogram, endoscopy, and manometry. The indications for each test depend on individual patient considerations and logistics specific to different clinical settings (2).

However, dysphagia remains an underdiagnosed condition. This is partly due to the lack of knowledge of the ICD code for dysphagia, meaning that many clinicians do not properly report the symptom at the time of taking the patient's clinical history. This often creates a negative cycle, where a patient is readmitted with a worsening condition that further limits the chances of determining an underlying problem with swallowing (25). Furthermore, there is a tendency to ‘normalise' the finding of cough in the older population, without performing an aetiological study of such symptoms (26). Consequently, the therapeutic approach to dysphagia tends to be poor or non-existent, with a subsequent impact that may inevitably result in the appearance of other disorders such as anxiety and depression (7).

As a result of this problem, different questionnaire-type tools have emerged to screen patients at risk for dysphagia in a simple and accessible way (7). Despite the development of such questionnaires, some tools are less practical when implemented, a factor that has a direct impact on their diagnostic capacity and real usefulness. For this reason, there is still a clinical need for an easy and quickly administered tool to screen for OD, its severity, and quality of life that can be used in any doctor's office regardless of the type of dysphagia presented (27).

**Figure 3:**
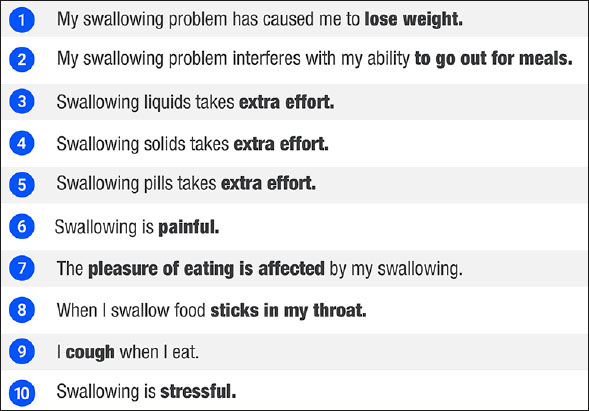
The Eating Assessment Tool-10 (EAT-10) questions (30)

One such tool was developed by Belafsky et al. (28): the Eating Assessment Tool-10 (EAT-10), which is a commonly used, quick, self-administered, and easy-to-understand survey-type instrument for the subjective assessment of dysphagia. A multidisciplinary team including gastroenterologists, otolaryngologists, nutritionists, and speech-language pathologists participated in the construction and validation of this instrument (27). The EAT-10 considers three domains – functional, emotional, and physical – that are evaluated in 10 questions (Figure 3) (29).

While the questions mostly assess difficulties in the swallowing process and thus help to evaluate OD (31), some of the questions could also help to assess gastro-oesophageal reflux disease (32).

Although the use of the EAT-10 in different clinical settings has been widely accepted due to its proven validity in various scenarios, its reliability, and its wide availability in different languages, it is critical to determine its real contribution in a clinical setting. Therefore, this work aims to provide a current overview of the EAT-10 to identify the lessons learned to date and its benefit in real clinical practice.

## Methodology

To identify aspects of the validation, applicability, and usefulness of EAT-10, a literature review was carried out among five electronic databases (Medline, Embase, Cochrane Database of Systematic Reviews, Cochrane Central, and LILACS) to obtain relevant, updated, and reliable evidence. Terms such as “EAT-10”, “validation” and “useful” were used for the search strategy. No restrictions were applied to language, date of publication, or study design; however, congress abstracts, posters, and studies that did not provide relevant information about the tool were excluded. In addition, a free search was conducted in the Google Scholar® platform complementary to the initial search in the aforementioned electronic databases. After the search, a screening was performed, followed by a full-text evaluation. Eligible references were subjected to a risk of bias assessment with tools validated for this purpose (Joanna Briggs Institute Critical Appraisal Tools and AMSTAR-2).

## Results

The list of included studies for analysis in this review is described in the table 1 (please see supplement 1 for further details on the studies) The overall quality of the obtained references was good, given the low risk of bias in most studies. The only studies that presented a high risk of bias were cross-sectional studies with a flawed methodological design; they have not been included in the results (see supplement 2 for the full risk of bias assessment). Although there are many remarkable aspects of the validation, usefulness, and applicability of the EAT-10 over time, the following list is a chronological sequence of the most relevant findings reported with regard to the EAT-10 among all publications.

**Table 1:** List of included studies in the analysis

**Author & year**	**Title**	**Study Design**	**Risk of bias**
Belafsky 2008	Validity and Reliability of the Eating Assessment Tool (EAT-10)	Cohort prospective Unicenter	Low
Burgos 2012	Translation and validation of the spanish version of the eating assessment tool-10 (EAT-10) for the screening of dysphagia	Cross sectional Prospective Multicenter	Low
Rebelo 2012	Cross-cultural adaptation of the Brazilian version of the Eating Assessment Tool – EAT-10	Cross sectional unicenter	High
Schindler 2013	Reliability and validity of the Italian Eating Assessment Tool	Cross sectional	Low
Cheney 2014	The Ability of the 10-Item Eating Assessment Tool (EAT-10) to Predict Aspiration Risk in Persons With Dysphagia	Cohort retrospective unicenter	Low
Mandysova 2014	Criterion validity of the self-report dysphagia assessment tool EAT-10 among neurological patients	Cross sectional Unicenter	High
Rofes 2014	Sensitivity and specificity of the Eating Assessment Tool and the Volume-Viscosity Swallow Test for clinical evaluation of oropharyngeal dysphagia	Cross sectional Unicenter	Low
Speyer 2014	Psychometric properties of questionnaires on functional health status in oropharyngeal dysphagia: A systematic literature review	Systematic Review	Moderate
Nogueira 2015	Measuring Outcomes for Dysphagia: Validity and Reliability of the European Portuguese Eating Assessment Tool (P-EAT-10)	Cohort Multicenter	Low
Wong 2015	Reliability and validity of the Chinese Eating Assessment Tool (EAT-10) in evaluation of acute stroke patients with dysphagia	Cross sectional Unicenter	Low
Arrese 2016	Relationship Between the Eating Assessment Tool-10 and Objective Clinical Ratings of Swallowing Function in Individuals with Head and Neck Cancer	Cross sectional Unicenter	Low
Cordier 2016	Evaluating the Psychometric Properties of the Eating Assessment Tool (EAT-10) Using Rasch Analysis	Rasch analysis (retrospective analysis with data from six hospitals)	Not applicable
Demir 2016	Reliability and Validity of the Turkish Eating Assessment Tool (T-EAT-10)	Cross sectional Unicenter	Low
Farahat 2016	Validation and Cultural Adaptation of the Arabic Version of the Eating Assessment Tool (EAT-10)	Cross sectional Unicenter	High
Giraldo-Cadavid 2016	Validation of the Spanish Version of the Eating Assessment Tool-10 (EAT-10spa) in Colombia. A Blinded Prospective Cohort Study	Cohort prospective Unicenter	Low
Leal 2016	Tradução Transcultural e Adaptação do “Eating Assessment Tool” para a Língua Portuguesa em Angola	Cross sectional Unicenter	High
Moller 2016	Validation of the Swedish translation of eating assessment tool (S-EAT-10)	Cohort prospective Unicenter	Low
Tenekeci 2016	Validity and Reliability of the Turkish Version of the Questionnaire for the Assessment of Dysphagia in Multiple Sclerosis	Cross sectional Unicenter	Low
Alali 2017	Dysphagia in Multiple Sclerosis: Evaluation and Validation of the DYMUS Questionnaire	Cross sectional	Low
Arslan 2017	The Ability of the Eating Assessment Tool-10 to Detect Aspiration in Patients With Neurological Disorders	Cross sectional	Low
Regan 2017	The Eating Assessment Tool-10 Predicts Aspiration in Adults with Stable Chronic Obstructive Pulmonary Disease	Cross sectional Unicenter	Low
Villamayor 2017	Prevalence of oropharyngeal dysphagia in an internal medicine unit and assessment of the utility of the Eating Assessment Tool 10 test in the routine evaluation	Cross sectional Unicenter	Low
Fernández-Rosati 2018	Validation of the eat-10 score to detect dysphagia in older people	Cross sectional Multicenter	Low
Mañas-Martínez 2018	Association of positive screening for dysphagia with nutritional status and long-term mortality in hospitalized elderly patients	Retro-spective Cohort Unicenter	Low
Printza 2018	Reliability and validity of the Eating Assessment Tool-10 (Greek adaptation) in neurogenic and head and neck cancer-related oropharyngeal dysphagia	Cohort prospective Unicenter	Low
Zaretsky 2018	Validation of the German version of Eating Assessment Tool for head and neck cancer patients	Cross sectional Unicenter	Low
Chung 2019	Validation of the Dutch EAT-10 for screening of oropharyngeal dysphagia in the elderly population	Cross sectional Unicenter	Low
Finger 2019	Analysis of the Prevalence and Onset of Dysphonia and Dysphagia Symptoms in Movement Disorders at an Academic Medical Center	Cross sectional Unicenter	Moderate
Lechien 2019	Validity and reliability of the French version of Eating Assessment Tool (EAT-10)	Cross sectional Bicenter	Low
Shapira-Galitz 2019	Does the Hebrew Eating Assessment Tool 10 Correlate with Pharyngeal Residue, Penetration and Aspiration on Fiberoptic Endoscopic Examination of Swallowing?	Cross sectional Bicenter	Low
Wilmskoetter 2019	Construct validity of the Eating Assessment Tool EAT-10	Rasch analysis (Retrospective analysis from a cohort)	Not applicable
Bofill-Soler 2020	Is EAT-10 Useful to Assess Swallowing during the Chemo-Radiotherapy Phase in Patients with Head and Neck Cancer? A Pilot Study	Cohort Prospective	Low
Casariego 2020	Utility of the EAT-10 in the detection of dysphagia in high-risk hospitalisation units at a university hospital: a cross-sectional study	Cross sectional Unicenter	Low
Hansen 2020	Item analysis of the Eating Assessment Tool (EAT-10) by the Rasch model: A secondary analysis of cross-sectional survey data obtained among community-dwelling elders	Rasch analysis (cross sectional data)	Not applicable
Krishnamurthy 2020	Evaluating the Psychometric Properties of the Kannada Version of EAT 10	Cross sectional Bicenter	Low
Lechien 2020	Validity and reliability of a French version of M.D. Anderson Dysphagia Inventory	Cross sectional Bicenter	Low
Möller 2020	A prospective study for evaluation of structural and clinical validity of the Eating Assessment Tool	Cross sectional Unicenter	Low
Printza 2020	The modified DYMUS questionnaire is a reliable, valid and easy-to-use tool in the assessment of dysphagia in multiple sclerosis	Cross sectional Unicenter	Low
Schlickewei 2020	The ability of the eating assessment tool 10 to detect penetration and aspiration in Parkinson's disease	Cross sectional	Low
Bartlett 2021	Correlation Between EAT 10 and Aspiration Risk Differs by Dysphagia Etiology	Cross sectional retrospective	Low
Dagna 2021	From DYMUS to DYPARK: Validation of a Screening Questionnaire for Dysphagia in Parkinson's Disease	Cross sectional Multicenter	Low
De Sire 2021	Screening dysphagia risk in 534 older patients undergoing rehabilitation after total joint replacement: a cross-sectional study	Cross sectional Unicenter	Low
Florie 2021	EAT-10 Scores and Fiberoptic Endoscopic Evaluation of Swallowing in Head and Neck Cancer Patients	Cross sectional Unicenter	Low
Pizzorni 2021	Dysphagia symptoms in obstructive sleep apnea: prevalence and clinical correlates	Prospective Inicenter	Low
Umay 2021	Esophageal dysphagia in neuromuscular disorder patients with validity and reliability study of the brief esophageal dysphagia questionnaire	Cross sectional Bicenter	Low
Yang 2021	Cultural Adaptation and Validation of Questionnaires for Evaluation of Health Related Quality of Life with Dysphagia in Different Countries: A Systematic Review	Systematic Review	Moderate

The EAT-10 was first published by Belafsky et al. (25) in 2008 in response to the need for an instrument that could assess OD in terms of severity, impact on quality of life, and effectiveness in a quick and easy way. After reliability and validity assessments, the authors concluded that the tool demonstrated adequate internal consistency, test-retest reproducibility, and criterion validity. The authors suggested defining an overall cut-off score of ≥ 3 as abnormal and recommended the presence of dysphagia be reported along with its initial severity and response to treatment in patients with different swallowing disorders (27).

By 2012, transcultural adaptions and language translations of the EAT-10 to Spanish and Portugues had been published. The authors highlighted that the EAT-10 is easy to use, easy to understand, and quickly answered by patients. This supports its usefulness when screening for dysphagia in individual practice, excluding populations with cognitive impairment (28). Researchers also noted that by not requiring formulas or visual analogue measurements to estimate the final score, the EAT-10 could potentially be used by a wide variety of health care professionals. This could contribute to the early identification of the need for intervention, decrease treatment cost, and improve quality of life (29).

In 2013, the Italian version of the EAT-10 was published. The authors reported a mild correlation between the EAT-10 score and the Flexible Endoscopic Evaluation of Swallowing (FEES) score. In the same study, the authors showed that the EAT-10 could estimate improvements after swallowing rehabilitation: their EAT-10 score decreased from 9.8 to 5.8. The authors noted that all patients completed the EAT-10 questionnaire in 4 minutes or less, and they recommended clinicians use this tool for everyday clinical practice and as a follow-up after treatment (33).

By 2014, in addition to its original objective, the EAT-10 had been tested to determine its usefulness in different settings. In one study, the authors evaluated the ability of the EAT-10 to screen for aspiration risk in patients with dysphagia and found a linear correlation between the EAT-10 score and the Penetration Aspiration Scale (PAS) score. This suggests that some of the subjective symptoms identified by the EAT-10 could also predict aspiration risk. With these findings, the researchers encouraged the standardisation of dysphagia screening protocols to reduce the incidence of aspiration pneumonia (34).

In 2014, Rofes et al. (35) reported adequate psychometric properties of the EAT-10 and the Volume-Viscosity Swallow Test (V-VST) for the clinical assessment of patients with OD assessed with a videofluoroscopic swallow study. The V-VST showed sensitivity of 94% and specificity of 88% for OD. Due to its high accuracy with a proposed cut-off score of 2 (with sensitivity of 89% and specificity of 82%), the authors suggested that the EAT-10 should be used in populations at risk for malnutrition and pneumonia to improve patient identification. Furthermore, the authors highlighted that dysphagia screening should be a fast, easy, and low-cost process and recommended that any patient with an EAT-10 score ≥ 2 should be considered for additional clinical dysphagia assessment (35).

In 2016, Giraldo-Cadavid et al. (36) also suggested a cutoff score of 2 to avoid missing any patients with dysphagia, instead of using the traditional cut-off score of 3. However, a score of 3 could be used as a cut-off when a better sensitivity/ specificity balance is desired. Their transcultural adaptation of the EAT-10 also detected changes in dysphagia severity due to improvement after treatment (sensitivity to change). The authors recommended that the tool be used as an aid in the early detection of dysphagia, to identify changes in severity and patients who require further assessment (36).

The EAT-10 has also been shown to be useful in testing the psychometric properties of other tools. For the Turkish adaptation of the Questionnaire for the Assessment of Dysphagia in Multiple Sclerosis (DYMUS), the researchers used the EAT-10 to test its criterion validity. The relationship between both tools was positive and statistically significant. Ultimately, the Turkish adaptation of the DYMUS was reliable and valid for dysphagia evaluation in patients with multiple sclerosis (37).

In 2016, Demir et al. (38) reported the validity and reliability of the Turkish version of the EAT-10 in a population with swallowing difficulties. The authors reported a mean time to complete the EAT-10 assessment of 1.8 ± 0.9 minutes.

In 2017, Arrese et al. (39) evaluated the relationship between the EAT-10 score and an objective clinical rating of swallowing physiology by using a modified barium swallow impairment profile (MBSImPTM) in patients with head and neck cancer. The mean EAT-10 score was significantly higher in patients with unsafe swallowing compared with those with safe swallowing (24 vs 16). The authors reported a correlation between the EAT-10 score and the MBSImPTM and PAS scores (39).

Also in 2017, Regan et al. (40) studied the correlation between the EAT-10 score and risk of aspiration in patients with stable chronic obstructive pulmonary disease (COPD). By considering a cut-off score of 9, the authors found that the EAT-10 could predict aspiration risk in this population. They suggested that the tool could aid in the early identification of patients at risk of dysphagia and aspiration, and thus help prevent clinically derived complications and hospital admissions in this population (40).

Zaretsky et al. (41) published the German adaptation of the EAT-10 in 2018. They validated the adapted tool in patients with head and neck cancer. They found positive associations between the EAT-10 and the body mass index (BMI), tumour stage, tumour location, and oncological therapy. The authors concluded that this adapted version is a reliable and valid questionnaire to assess possible dysphagia in this population (41).

On the other hand, possible flaws of the EAT-10 tool have been addressed in aspects like structural validity, internal consistency, and item redundancy. Taking into account a Rasch analysis approach, the EAT-10 psychometric properties, usefulness, and applicability could be improved by addressing those flaws. Moreover, it may not be suitable for use in certain populations, such as individuals with cognitive impairment or severe psychiatric conditions, where its utility as a self-administered tool may be restricted by difficulties in understanding the questions and limitations in providing self-reports of symptoms. This may ultimately affect the accuracy and validity of the tool results (21). However, clinical experience has shown that instruments like the EAT-10 help clinicians and patients reach a common understanding of the patient's swallowing complaints. This allowing for opportunities for targeted treatment options that address individualised goals (42, 43).

The EAT-10 has also been used as an instrument for prevalence studies. In 2021. Pizzorni et al. (44) analysed the prevalence of OD in 951 patients with obstructive sleep apnoea. They found that 141 patients reported an EAT-10 score of > 3. In multivariate analyses, EAT-10 scores were associated with gender, excessive daily sleepiness, the number of obstructive sleep apnoea symptoms, anxiety/depression, and symptoms of gastro-oesophageal reflux.

Regarding the discriminatory ability of the EAT-10 in specific diseases such as amyotrophic lateral sclerosis, studies like those by Plowman and Donohue (45, 46) have found that the tool has a good discriminatory ability to identify patients with a non-safe airway during the swallowing process. They have concluded that it is an appropriate and safe swallowing assessment tool. Thus, it could aid in OD screening in these patients, facilitating prompt further evaluation and treatment.

In a recently published systematic review and meta-analysis, the authors assessed the diagnostic accuracy of the EAT-10 cutoff score by using FEES or a videofluoroscopic swallow study as the gold standard (47). The areas under the curve were 0.873 (95% confidence interval [CI] 0.82–0.93) and 0.903 (95% CI 0.88–0.93) for cut-off scores of 2 and 3, respectively. Although these results are good, a cut-off value of 3 is still recommended for better diagnostic accuracy.

## Discussion

The high rate of OD underdiagnosis makes the EAT-10 an optimal instrument to be used in clinical practice to select patients at risk of dysphagia. Because of its applicability and accessibility, it could be used in different settings such as hospitals, nursing homes, and external consultation all over the world. Patients who fail their EAT-10 assessment should be referred for further clinical and instrumental assessment.

### Benefits in Real Clinical Practice

When considering the impact of the EAT-10 on real clinical practice, in combination with emerging evidence published every year, it is clear that standardising screening protocols for dysphagia is a good strategy to improve the diagnosis of OD, a condition that remains neglected. Indeed, studies by authors such as Sherman et al. (48) have highlighted that early screening for dysphagia can positively impact the prognosis of patients with acute stroke by reducing the risk of complications such as pneumonia, dependency, prolonged hospital stays, and even death. Therefore, it is not surprising that some authors have suggested using the EAT-10 for selective screening in the older population at high risk of dysphagia, even if this was not its originally intended purpose.

Furthermore, the EAT-10 has been described as a suitable screening tool for dysphagia based on its observed characteristics, such as easy application and interpretation, quick completion, and low cost. These features make it suitable for non-specialised health care providers to screen for OD.

In terms of its applicability in clinical practice, the EAT-10 has been correlated with other clinical parameters, especially in evaluating aspiration risk. Studies have identified a correlation between the EAT-10 score and aspiration (specifically with the PAS score) and, subsequently, identified patients at risk of pneumonia, malnutrition, or even death.

The validity of the EAT-10 has also been demonstrated in its ability to predict aspiration when administered to patients with dysphagia and significant comorbidities like COPD, head and neck cancer, obstructive sleep apnoea, and neurological disorders. Furthermore, the EAT-10 has been used as a reference standard for the validation of other scales and tools, most notably the DYMUS (37, 49, 50, 51). This suggests that, based on its wide availability and demonstrated reliability, clinicians and researchers are beginning to adopt the EAT-10 as a reference standard.

These findings demonstrate that the EAT-10 could have a direct impact on reducing complications, as well as in the reduction of economic and social costs derived from those complications (31, 35). Therefore, the EAT-10 could be used either in the development of dysphagia screening protocols, or in preventative protocols seeking to reduce the incidence of dysphagia-related complications. These protocols also have the potential to help determine whether further assessment is needed in any given patient.

### Significant Features of the EAT-10

Clinicians and other researchers have advocated for the use of the EAT-10 as a viable screening tool for dysphagia, although there is some disagreement about its use (42). They have argued that this tool could also be useful in identifying patients who require a more comprehensive assessment (40). As discussed by Alali et al. (51), psychometric validation is not the only factor one needs to consider when using an assessment tool. Other important considerations include clinical experience and user feedback, as well as its simplicity in terms of use.

In this regard, the EAT-10 represents a useful follow-up tool based on its quick completion time (3–4 minutes), that fact that the final score is determined without formulas or scales, and its sensitivity to changes in dysphagia severity before and after treatment. Furthermore, the EAT-10 scoring system is versatile, a factor that could provide flexibility depending on clinical, population, and research needs. This has been demonstrated by some authors who have proposed cut-off scores different from the original cut-off score of 3 defined by Belafsky et al. 25).

Lastly, the EAT-10 has been used as an aid to determine the prevalence of OD in several studies (44, 52, 53, 54, 55). Because the concept and definition of dysphagia itself varies greatly among sources, authors could establish different definitions for the purposes of their studies (i.e., one group of authors could consider dysphagia as any difficulty in the swallow process, while another group could consider dysphagia only when it affects quality of life), thereby making it difficult to estimate prevalence accurately. However, the EAT-10 could provide a standardised way of reporting and identifying the prevalence of OD.

### Special Considerations Regarding Adaptation of the EAT-10 to Different Countries

**Figure 4:**
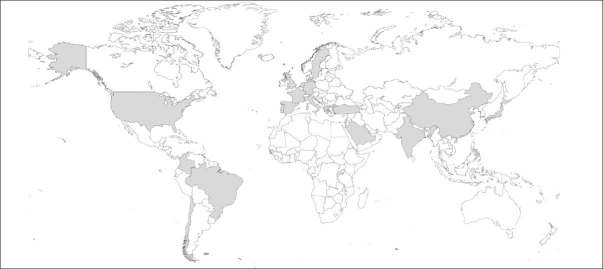
Worldwide availability of the Eating Assessment Tool-10 (EAT-10) adaptations

The EAT-10 has undergone numerous transcultural adaptations and language translations. In each case, the psychometric properties of the EAT-10 have been tested and produced good overall results, thus encouraging its use in real clinical practice. Therefore, the EAT-10 could aid in identifying the risk of dysphagia not only at a national level, but also on a global scale, increasing the availability of data and feedback regarding its use. As of 2022, over 15 translations, cultural adaptations, and validations have been developed for the EAT-10: Spanish (Spain, Chile, and Colombia), French, Italian, Portuguese (European and Brazilian), German, Chinese, Japanese, Arabic, Dutch, Greek, Hebrew, Swedish, and Turkish (25, 29, 32, 34, 37, 51, 52, 53, 54, 55, 56, 57, 58, 59). A world map of the territorial coverage of these adaptations is shown in Figure 4.

## Conclusions

### Practical Considerations

Dysphagia is an under-diagnosed but potentially serious condition: it could imply a high risk of serious clinical complications that even lead to the death of patients with specific phenotypes. Thus, screening for dysphagia is mandatory as a first step for diagnosis.

**Figure 5:**
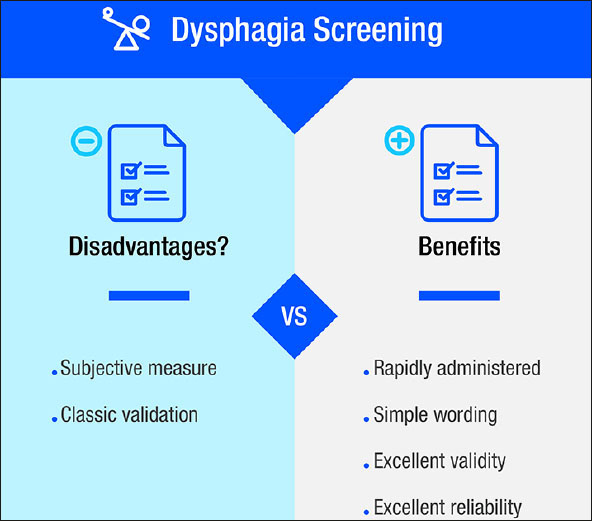
The benefits of the Eating Assessment Tool-10 (EAT-10) outweigh its potential disadvantages

The EAT-10 has proved to be a viable tool in screening for and identifying OD, based on its reported reliability and validity in different settings across different phenotypes of patients with dysphagia, languages, and health conditions. Key features include a quick completion time, easy applicability, a simple scoring system, and low cost; the benefits of the EAT-10 outweigh its potential disadvantages (Figure 5).

The possibility of using different cut-off scores of the EAT-10 has also been studied. This ability could allow the tool to be applied to different populations as well as for different clinical or research needs.

The universal application of EAT-10 among at-risk populations, together with its excellent psychometric properties, will improve the identification of patients with OD at risk for malnutrition and aspiration pneumonia. The feedback and recommendations provided by all health care providers, authors, and researchers thanks to the numerous adaptations and translations of the EAT-10 –allowing for its use in multiple languages and cultures– could further improve the screening, diagnosis, and follow-up for patients with oropharyngeal dysphagia.

## Electronic Supplementary Material

Risk of bias assessment in included studies
