# Supplementary material for: History and Science behind the Eating Assessment Tool-10 (Eat-10): Lessons Learned
Source: J Nutr Health Aging. 2023 Jul 28;27(8):597–606. doi: 10.1007/s12603-023-1950-9 (PMC12877663; doi:10.1007/s12603-023-1950-9)
Supplement: Supplementary file 1 — Risk of bias assessment in included studies [file mmc1.docx]

# Supplement

# Risk of bias assessment in included studies

## Cross-sectional studies

| **JBI Critical Appraisal Checklist for Cross-sectional Studies** | | | |  |
| --- | --- | --- | --- | --- |
|  |  |  |  |  |
| **Title:** | Correlation Between EAT‑10 and Aspiration Risk Differs by Dysphagia Etiology | | |  |
|  |  |  |  |  |
| **Author:** | Bartlett | **Year:** | 2021 |  |
| **#** | **Question** | | **Outcome** |  |
| **1** | Were the criteria for inclusion in the sample clearly defined? | | Yes |  |
|  |  |  |  |  |
| **2** | Were the study subjects and the setting described in detail? | | Yes |  |
|  |  |  |  |  |
| **3** | Was the exposure measured in a valid and reliable way? | | Yes |  |
|  |  |  |  |  |
| **4** | Were objective, standard criteria used for measurement of the condition? | | Yes |  |
|  |  |  |  |  |
| **5** | Were confounding factors identified? | | Yes |  |
|  |  |  |  |  |
| **6** | Were strategies to deal with confounding factors stated? | | Yes |  |
|  |  |  |  |  |
| **7** | Were the outcomes measured in a valid and reliable way? | | Yes |  |
|  |  |  |  |  |
| **8** | Was appropriate statistical analysis used? | | Yes |  |
|  |  |  |  |  |

| **JBI Critical Appraisal Checklist for Cross-sectional Studies** | | | |  |
| --- | --- | --- | --- | --- |
|  |  |  |  |  |
| **Title:** | From DYMUS to DYPARK: Validation of a Screening Questionnaire for Dysphagia in Parkinson's Disease | | |  |
|  |  |  |  |  |
| **Author:** | Dagna | **Year:** | 2021 |  |
| **#** | **Question** | | **Outcome** |  |
| **1** | Were the criteria for inclusion in the sample clearly defined? | | Yes |  |
|  |  |  |  |  |
| **2** | Were the study subjects and the setting described in detail? | | Yes |  |
|  |  |  |  |  |
| **3** | Was the exposure measured in a valid and reliable way? | | Yes |  |
|  |  |  |  |  |
| **4** | Were objective, standard criteria used for measurement of the condition? | | Yes |  |
|  |  |  |  |  |
| **5** | Were confounding factors identified? | | Not applicable |  |
|  |  |  |  |  |
| **6** | Were strategies to deal with confounding factors stated? | | Not applicable |  |
|  |  |  |  |  |
| **7** | Were the outcomes measured in a valid and reliable way? | | Yes |  |
|  |  |  |  |  |
| **8** | Was appropriate statistical analysis used? | | Yes |  |
|  |  |  |  |  |

| **JBI Critical Appraisal Checklist for Cross-sectional Studies** | | | |  |
| --- | --- | --- | --- | --- |
|  |  |  |  |  |
| **Title:** | Screening dysphagia risk in 534 older patients undergoing rehabilitation after total joint replacement: a cross-sectional study | | |  |
|  |  |  |  |  |
| **Author:** | De Sire | **Year:** | 2021 |  |
| **#** | **Question** | | **Outcome** |  |
| **1** | Were the criteria for inclusion in the sample clearly defined? | | Yes |  |
|  |  |  |  |  |
| **2** | Were the study subjects and the setting described in detail? | | Yes |  |
|  |  |  |  |  |
| **3** | Was the exposure measured in a valid and reliable way? | | Yes |  |
|  |  |  |  |  |
| **4** | Were objective, standard criteria used for measurement of the condition? | | Yes |  |
|  |  |  |  |  |
| **5** | Were confounding factors identified? | | Yes |  |
|  |  |  |  |  |
| **6** | Were strategies to deal with confounding factors stated? | | Yes |  |
|  |  |  |  |  |
| **7** | Were the outcomes measured in a valid and reliable way? | | Yes |  |
|  |  |  |  |  |
| **8** | Was appropriate statistical analysis used? | | Yes |  |
|  |  |  |  |  |

| **JBI Critical Appraisal Checklist for Cross-sectional Studies** | | | |  |
| --- | --- | --- | --- | --- |
|  |  |  |  |  |
| **Title:** | EAT-10 Scores and Fiberoptic Endoscopic Evaluation of Swallowing in Head and Neck Cancer Patients | | |  |
|  |  |  |  |  |
| **Author:** | Florie | **Year:** | 2021 |  |
| **#** | **Question** | | **Outcome** |  |
| **1** | Were the criteria for inclusion in the sample clearly defined? | | Yes |  |
|  |  |  |  |  |
| **2** | Were the study subjects and the setting described in detail? | | Yes |  |
|  |  |  |  |  |
| **3** | Was the exposure measured in a valid and reliable way? | | Yes |  |
|  |  |  |  |  |
| **4** | Were objective, standard criteria used for measurement of the condition? | | Yes |  |
|  |  |  |  |  |
| **5** | Were confounding factors identified? | | Yes |  |
|  |  |  |  |  |
| **6** | Were strategies to deal with confounding factors stated? | | Yes |  |
|  |  |  |  |  |
| **7** | Were the outcomes measured in a valid and reliable way? | | Yes |  |
|  |  |  |  |  |
| **8** | Was appropriate statistical analysis used? | | Yes |  |
|  |  |  |  |  |

| **JBI Critical Appraisal Checklist for Cross-sectional Studies** | | | |  |
| --- | --- | --- | --- | --- |
|  |  |  |  |  |
| **Title:** | Esophageal dysphagia in neuromuscular disorder patients with validity and reliability study of the brief esophageal dysphagia questionnaire | | |  |
|  |  |  |  |  |
| **Author:** | Umay | **Year:** | 2021 |  |
| **#** | **Question** | | **Outcome** |  |
| **1** | Were the criteria for inclusion in the sample clearly defined? | | Yes |  |
|  |  |  |  |  |
| **2** | Were the study subjects and the setting described in detail? | | Yes |  |
|  |  |  |  |  |
| **3** | Was the exposure measured in a valid and reliable way? | | Yes |  |
|  |  |  |  |  |
| **4** | Were objective, standard criteria used for measurement of the condition? | | Yes |  |
|  |  |  |  |  |
| **5** | Were confounding factors identified? | | Yes |  |
|  |  |  |  |  |
| **6** | Were strategies to deal with confounding factors stated? | | Yes |  |
|  |  |  |  |  |
| **7** | Were the outcomes measured in a valid and reliable way? | | Yes |  |
|  |  |  |  |  |
| **8** | Was appropriate statistical analysis used? | | Yes |  |
|  |  |  |  |  |

| **JBI Critical Appraisal Checklist for Cross-sectional Studies** | | | |  |
| --- | --- | --- | --- | --- |
|  |  |  |  |  |
| **Title:** | Utility of the EAT-10 in the detection of dysphagia in high-risk hospitalisation units at a university hospital: a cross-sectional study | | |  |
|  |  |  |  |  |
| **Author:** | Casadiego | **Year:** | 2020 |  |
| **#** | **Question** | | **Outcome** |  |
| **1** | Were the criteria for inclusion in the sample clearly defined? | | Yes |  |
|  |  |  |  |  |
| **2** | Were the study subjects and the setting described in detail? | | Yes |  |
|  |  |  |  |  |
| **3** | Was the exposure measured in a valid and reliable way? | | Yes |  |
|  |  |  |  |  |
| **4** | Were objective, standard criteria used for measurement of the condition? | | Yes |  |
|  |  |  |  |  |
| **5** | Were confounding factors identified? | | Yes |  |
|  |  |  |  |  |
| **6** | Were strategies to deal with confounding factors stated? | | Yes |  |
|  |  |  |  |  |
| **7** | Were the outcomes measured in a valid and reliable way? | | Yes |  |
|  |  |  |  |  |
| **8** | Was appropriate statistical analysis used? | | Yes |  |
|  |  |  |  |  |

| **JBI Critical Appraisal Checklist for Cross-sectional Studies** | | | |  |
| --- | --- | --- | --- | --- |
|  |  |  |  |  |
| **Title:** | Evaluating the Psychometric Properties of the Kannada Version of EAT 10 | | |  |
|  |  |  |  |  |
| **Author:** | Krishnamurthy | **Year:** | 2020 |  |
| **#** | **Question** | | **Outcome** |  |
| **1** | Were the criteria for inclusion in the sample clearly defined? | | Yes |  |
|  |  |  |  |  |
| **2** | Were the study subjects and the setting described in detail? | | Yes |  |
|  |  |  |  |  |
| **3** | Was the exposure measured in a valid and reliable way? | | Yes |  |
|  |  |  |  |  |
| **4** | Were objective, standard criteria used for measurement of the condition? | | Yes |  |
|  |  |  |  |  |
| **5** | Were confounding factors identified? | | No |  |
|  |  |  |  |  |
| **6** | Were strategies to deal with confounding factors stated? | | No |  |
|  |  |  |  |  |
| **7** | Were the outcomes measured in a valid and reliable way? | | Yes |  |
|  |  |  |  |  |
| **8** | Was appropriate statistical analysis used? | | Yes |  |
|  |  |  |  |  |

| **JBI Critical Appraisal Checklist for Cross-sectional Studies** | | | |  |
| --- | --- | --- | --- | --- |
|  |  |  |  |  |
| **Title:** | Validity and reliability of a French version of M.D. Anderson Dysphagia Inventory | | |  |
|  |  |  |  |  |
| **Author:** | Lechien | **Year:** | 2020 |  |
| **#** | **Question** | | **Outcome** |  |
| **1** | Were the criteria for inclusion in the sample clearly defined? | | Yes |  |
|  |  |  |  |  |
| **2** | Were the study subjects and the setting described in detail? | | Yes |  |
|  |  |  |  |  |
| **3** | Was the exposure measured in a valid and reliable way? | | Yes |  |
|  |  |  |  |  |
| **4** | Were objective, standard criteria used for measurement of the condition? | | Yes |  |
|  |  |  |  |  |
| **5** | Were confounding factors identified? | | Yes |  |
|  |  |  |  |  |
| **6** | Were strategies to deal with confounding factors stated? | | Yes |  |
|  |  |  |  |  |
| **7** | Were the outcomes measured in a valid and reliable way? | | Yes |  |
|  |  |  |  |  |
| **8** | Was appropriate statistical analysis used? | | Yes |  |
|  |  |  |  |  |

| **JBI Critical Appraisal Checklist for Cross-sectional Studies** | | | |  |
| --- | --- | --- | --- | --- |
|  |  |  |  |  |
| **Title:** | A prospective study for evaluation of structural and clinical validity of the  Eating Assessment Tool | | |  |
|  |  |  |  |  |
| **Author:** | Möller | **Year:** | 2020 |  |
| **#** | **Question** | | **Outcome** |  |
| **1** | Were the criteria for inclusion in the sample clearly defined? | | Yes |  |
|  |  |  |  |  |
| **2** | Were the study subjects and the setting described in detail? | | Yes |  |
|  |  |  |  |  |
| **3** | Was the exposure measured in a valid and reliable way? | | Yes |  |
|  |  |  |  |  |
| **4** | Were objective, standard criteria used for measurement of the condition? | | Yes |  |
|  |  |  |  |  |
| **5** | Were confounding factors identified? | | Yes |  |
|  |  |  |  |  |
| **6** | Were strategies to deal with confounding factors stated? | | Yes |  |
|  |  |  |  |  |
| **7** | Were the outcomes measured in a valid and reliable way? | | Yes |  |
|  |  |  |  |  |
| **8** | Was appropriate statistical analysis used? | | Yes |  |
|  |  |  |  |  |

| **JBI Critical Appraisal Checklist for Cross-sectional Studies** | | | |  |
| --- | --- | --- | --- | --- |
|  |  |  |  |  |
| **Title:** | The modified DYMUS questionnaire is a reliable, valid and easy-to-use tool in the assessment of dysphagia in multiple sclerosis | | |  |
|  |  |  |  |  |
| **Author:** | Printza | **Year:** | 2020 |  |
| **#** | **Question** | | **Outcome** |  |
| **1** | Were the criteria for inclusion in the sample clearly defined? | | Yes |  |
|  |  |  |  |  |
| **2** | Were the study subjects and the setting described in detail? | | Yes |  |
|  |  |  |  |  |
| **3** | Was the exposure measured in a valid and reliable way? | | Yes |  |
|  |  |  |  |  |
| **4** | Were objective, standard criteria used for measurement of the condition? | | Yes |  |
|  |  |  |  |  |
| **5** | Were confounding factors identified? | | Yes |  |
|  |  |  |  |  |
| **6** | Were strategies to deal with confounding factors stated? | | Yes |  |
|  |  |  |  |  |
| **7** | Were the outcomes measured in a valid and reliable way? | | Yes |  |
|  |  |  |  |  |
| **8** | Was appropriate statistical analysis used? | | Yes |  |
|  |  |  |  |  |

| **JBI Critical Appraisal Checklist for Cross-sectional Studies** | | | |  |
| --- | --- | --- | --- | --- |
|  |  |  |  |  |
| **Title:** | The ability of the eating assessment tool‑10 to detect penetration and aspiration in Parkinson’s disease | | |  |
|  |  |  |  |  |
| **Author:** | Schlickewei | **Year:** | 2020 |  |
| **#** | **Question** | | **Outcome** |  |
| **1** | Were the criteria for inclusion in the sample clearly defined? | | Yes |  |
|  |  |  |  |  |
| **2** | Were the study subjects and the setting described in detail? | | Yes |  |
|  |  |  |  |  |
| **3** | Was the exposure measured in a valid and reliable way? | | Yes |  |
|  |  |  |  |  |
| **4** | Were objective, standard criteria used for measurement of the condition? | | Yes |  |
|  |  |  |  |  |
| **5** | Were confounding factors identified? | | Yes |  |
|  |  |  |  |  |
| **6** | Were strategies to deal with confounding factors stated? | | Yes |  |
|  |  |  |  |  |
| **7** | Were the outcomes measured in a valid and reliable way? | | Yes |  |
|  |  |  |  |  |
| **8** | Was appropriate statistical analysis used? | | Yes |  |
|  |  |  |  |  |

| **JBI Critical Appraisal Checklist for Cross-sectional Studies** | | | |  |
| --- | --- | --- | --- | --- |
|  |  |  |  |  |
| **Title:** | Validation of the Dutch EAT-10 for screening of oropharyngeal dysphagia in the elderly population | | |  |
|  |  |  |  |  |
| **Author:** | Chung | **Year:** | 2019 |  |
| **#** | **Question** | | **Outcome** |  |
| **1** | Were the criteria for inclusion in the sample clearly defined? | | Yes |  |
|  |  |  |  |  |
| **2** | Were the study subjects and the setting described in detail? | | Yes |  |
|  |  |  |  |  |
| **3** | Was the exposure measured in a valid and reliable way? | | Yes |  |
|  |  |  |  |  |
| **4** | Were objective, standard criteria used for measurement of the condition? | | Yes |  |
|  |  |  |  |  |
| **5** | Were confounding factors identified? | | Not applicable |  |
|  |  |  |  |  |
| **6** | Were strategies to deal with confounding factors stated? | | Not applicable |  |
|  |  |  |  |  |
| **7** | Were the outcomes measured in a valid and reliable way? | | Yes |  |
|  |  |  |  |  |
| **8** | Was appropriate statistical analysis used? | | Yes |  |
|  |  |  |  |  |

| **JBI Critical Appraisal Checklist for Cross-sectional Studies** | | | |  |
| --- | --- | --- | --- | --- |
|  |  |  |  |  |
| **Title:** | Analysis of the Prevalence and Onset of Dysphonia and Dysphagia Symptoms in Movement Disorders at an Academic Medical Center | | |  |
|  |  |  |  |  |
| **Author:** | Finger | **Year:** | 2019 |  |
| **#** | **Question** | | **Outcome** |  |
| **1** | Were the criteria for inclusion in the sample clearly defined? | | Yes |  |
|  |  |  |  |  |
| **2** | Were the study subjects and the setting described in detail? | | Yes |  |
|  |  |  |  |  |
| **3** | Was the exposure measured in a valid and reliable way? | | Yes |  |
|  |  |  |  |  |
| **4** | Were objective, standard criteria used for measurement of the condition? | | Yes |  |
|  |  |  |  |  |
| **5** | Were confounding factors identified? | | Not applicable |  |
|  |  |  |  |  |
| **6** | Were strategies to deal with confounding factors stated? | | Not applicable |  |
|  |  |  |  |  |
| **7** | Were the outcomes measured in a valid and reliable way? | | Yes |  |
|  |  |  |  |  |
| **8** | Was appropriate statistical analysis used? | | Yes |  |
|  |  |  |  |  |

| **JBI Critical Appraisal Checklist for Cross-sectional Studies** | | | |  |
| --- | --- | --- | --- | --- |
|  |  |  |  |  |
| **Title:** | Validity and reliability of the French version of Eating Assessment Tool (EAT-10) | | |  |
|  |  |  |  |  |
| **Author:** | Lechien | **Year:** | 2019 |  |
| **#** | **Question** | | **Outcome** |  |
| **1** | Were the criteria for inclusion in the sample clearly defined? | | Yes |  |
|  |  |  |  |  |
| **2** | Were the study subjects and the setting described in detail? | | Yes |  |
|  |  |  |  |  |
| **3** | Was the exposure measured in a valid and reliable way? | | Yes |  |
|  |  |  |  |  |
| **4** | Were objective, standard criteria used for measurement of the condition? | | Yes |  |
|  |  |  |  |  |
| **5** | Were confounding factors identified? | | Yes |  |
|  |  |  |  |  |
| **6** | Were strategies to deal with confounding factors stated? | | Yes |  |
|  |  |  |  |  |
| **7** | Were the outcomes measured in a valid and reliable way? | | Yes |  |
|  |  |  |  |  |
| **8** | Was appropriate statistical analysis used? | | Yes |  |
|  |  |  |  |  |

| **JBI Critical Appraisal Checklist for Cross-sectional Studies** | | | |  |
| --- | --- | --- | --- | --- |
|  |  |  |  |  |
| **Title:** | Does the Hebrew Eating Assessment Tool‑10 Correlate with Pharyngeal Residue, Penetration and Aspiration on Fiberoptic Endoscopic Examination of Swallowing? | | |  |
|  |  |  |  |  |
| **Author:** | Shapira-Galitz | **Year:** | 2019 |  |
| **#** | **Question** | | **Outcome** |  |
| **1** | Were the criteria for inclusion in the sample clearly defined? | | Yes |  |
|  |  |  |  |  |
| **2** | Were the study subjects and the setting described in detail? | | Yes |  |
|  |  |  |  |  |
| **3** | Was the exposure measured in a valid and reliable way? | | Yes |  |
|  |  |  |  |  |
| **4** | Were objective, standard criteria used for measurement of the condition? | | Yes |  |
|  |  |  |  |  |
| **5** | Were confounding factors identified? | | Yes |  |
|  |  |  |  |  |
| **6** | Were strategies to deal with confounding factors stated? | | Yes |  |
|  |  |  |  |  |
| **7** | Were the outcomes measured in a valid and reliable way? | | Yes |  |
|  |  |  |  |  |
| **8** | Was appropriate statistical analysis used? | | Yes |  |
|  |  |  |  |  |

| **JBI Critical Appraisal Checklist for Cross-sectional Studies** | | | |  |
| --- | --- | --- | --- | --- |
|  |  |  |  |  |
| **Title:** | Validation of the eat-10 score to detect dysphagia in older people | | |  |
|  |  |  |  |  |
| **Author:** | Fernández-Rosati | **Year:** | 2018 |  |
| **#** | **Question** | | **Outcome** |  |
| **1** | Were the criteria for inclusion in the sample clearly defined? | | Yes |  |
|  |  |  |  |  |
| **2** | Were the study subjects and the setting described in detail? | | Yes |  |
|  |  |  |  |  |
| **3** | Was the exposure measured in a valid and reliable way? | | Yes |  |
|  |  |  |  |  |
| **4** | Were objective, standard criteria used for measurement of the condition? | | Yes |  |
|  |  |  |  |  |
| **5** | Were confounding factors identified? | | Yes |  |
|  |  |  |  |  |
| **6** | Were strategies to deal with confounding factors stated? | | Yes |  |
|  |  |  |  |  |
| **7** | Were the outcomes measured in a valid and reliable way? | | Yes |  |
|  |  |  |  |  |
| **8** | Was appropriate statistical analysis used? | | Yes |  |
|  |  |  |  |  |

| **JBI Critical Appraisal Checklist for Cross-sectional Studies** | | | |  |
| --- | --- | --- | --- | --- |
|  |  |  |  |  |
| **Title:** | Validation of the German version of Eating Assessment Tool for head and neck cancer patients | | |  |
|  |  |  |  |  |
| **Author:** | Zaretsky | **Year:** | 2018 |  |
| **#** | **Question** | | **Outcome** |  |
| **1** | Were the criteria for inclusion in the sample clearly defined? | | Yes |  |
|  |  |  |  |  |
| **2** | Were the study subjects and the setting described in detail? | | Yes |  |
|  |  |  |  |  |
| **3** | Was the exposure measured in a valid and reliable way? | | Yes |  |
|  |  |  |  |  |
| **4** | Were objective, standard criteria used for measurement of the condition? | | Yes |  |
|  |  |  |  |  |
| **5** | Were confounding factors identified? | | Yes |  |
|  |  |  |  |  |
| **6** | Were strategies to deal with confounding factors stated? | | Yes |  |
|  |  |  |  |  |
| **7** | Were the outcomes measured in a valid and reliable way? | | Yes |  |
|  |  |  |  |  |
| **8** | Was appropriate statistical analysis used? | | Yes |  |
|  |  |  |  |  |

| **JBI Critical Appraisal Checklist for Cross-sectional Studies** | | | |  |
| --- | --- | --- | --- | --- |
|  |  |  |  |  |
| **Title:** | Dysphagia in Multiple Sclerosis: Evaluation and Validation of the DYMUS Questionnaire | | |  |
|  |  |  |  |  |
| **Author:** | Alali | **Year:** | 2017 |  |
| **#** | **Question** | | **Outcome** |  |
| **1** | Were the criteria for inclusion in the sample clearly defined? | | Yes |  |
|  |  |  |  |  |
| **2** | Were the study subjects and the setting described in detail? | | Yes |  |
|  |  |  |  |  |
| **3** | Was the exposure measured in a valid and reliable way? | | Yes |  |
|  |  |  |  |  |
| **4** | Were objective, standard criteria used for measurement of the condition? | | Yes |  |
|  |  |  |  |  |
| **5** | Were confounding factors identified? | | Yes |  |
|  |  |  |  |  |
| **6** | Were strategies to deal with confounding factors stated? | | Yes |  |
|  |  |  |  |  |
| **7** | Were the outcomes measured in a valid and reliable way? | | Yes |  |
|  |  |  |  |  |
| **8** | Was appropriate statistical analysis used? | | Yes |  |
|  |  |  |  |  |

| **JBI Critical Appraisal Checklist for Cross-sectional Studies** | | | |  |
| --- | --- | --- | --- | --- |
|  |  |  |  |  |
| **Title:** | The Ability of the Eating Assessment Tool-10 to Detect Aspiration in Patients With Neurological Disorders | | |  |
|  |  |  |  |  |
| **Author:** | Arslan | **Year:** | 2017 |  |
| **#** | **Question** | | **Outcome** |  |
| **1** | Were the criteria for inclusion in the sample clearly defined? | | Yes |  |
|  |  |  |  |  |
| **2** | Were the study subjects and the setting described in detail? | | No |  |
|  |  |  |  |  |
| **3** | Was the exposure measured in a valid and reliable way? | | Yes |  |
|  |  |  |  |  |
| **4** | Were objective, standard criteria used for measurement of the condition? | | Yes |  |
|  |  |  |  |  |
| **5** | Were confounding factors identified? | | No |  |
|  |  |  |  |  |
| **6** | Were strategies to deal with confounding factors stated? | | No |  |
|  |  |  |  |  |
| **7** | Were the outcomes measured in a valid and reliable way? | | Yes |  |
|  |  |  |  |  |
| **8** | Was appropriate statistical analysis used? | | Yes |  |
|  |  |  |  |  |

| **JBI Critical Appraisal Checklist for Cross-sectional Studies** | | | |  |
| --- | --- | --- | --- | --- |
|  |  |  |  |  |
| **Title:** | The Eating Assessment Tool-10 Predicts Aspiration in Adults with Stable Chronic Obstructive Pulmonary Disease | | |  |
|  |  |  |  |  |
| **Author:** | Regan | **Year:** | 2017 |  |
| **#** | **Question** | | **Outcome** |  |
| **1** | Were the criteria for inclusion in the sample clearly defined? | | Yes |  |
|  |  |  |  |  |
| **2** | Were the study subjects and the setting described in detail? | | No |  |
|  |  |  |  |  |
| **3** | Was the exposure measured in a valid and reliable way? | | Yes |  |
|  |  |  |  |  |
| **4** | Were objective, standard criteria used for measurement of the condition? | | Yes |  |
|  |  |  |  |  |
| **5** | Were confounding factors identified? | | Yes |  |
|  |  |  |  |  |
| **6** | Were strategies to deal with confounding factors stated? | | Yes |  |
|  |  |  |  |  |
| **7** | Were the outcomes measured in a valid and reliable way? | | Yes |  |
|  |  |  |  |  |
| **8** | Was appropriate statistical analysis used? | | Yes |  |
|  |  |  |  |  |

| **JBI Critical Appraisal Checklist for Cross-sectional Studies** | | | |  |
| --- | --- | --- | --- | --- |
|  |  |  |  |  |
| **Title:** | Prevalence of oropharyngeal dysphagia in an internal medicine unit and assessment of the utility of the Eating Assessment Tool 10 test in the routine evaluation | | |  |
|  |  |  |  |  |
| **Author:** | Villamayor | **Year:** | 2017 |  |
| **#** | **Question** | | **Outcome** |  |
| **1** | Were the criteria for inclusion in the sample clearly defined? | | Yes |  |
|  |  |  |  |  |
| **2** | Were the study subjects and the setting described in detail? | | No |  |
|  |  |  |  |  |
| **3** | Was the exposure measured in a valid and reliable way? | | Yes |  |
|  |  |  |  |  |
| **4** | Were objective, standard criteria used for measurement of the condition? | | Yes |  |
|  |  |  |  |  |
| **5** | Were confounding factors identified? | | Yes |  |
|  |  |  |  |  |
| **6** | Were strategies to deal with confounding factors stated? | | Not applicable |  |
|  |  |  |  |  |
| **7** | Were the outcomes measured in a valid and reliable way? | | Yes |  |
|  |  |  |  |  |
| **8** | Was appropriate statistical analysis used? | | Yes |  |
|  |  |  |  |  |

| **JBI Critical Appraisal Checklist for Cross-sectional Studies** | | | |  |
| --- | --- | --- | --- | --- |
|  |  |  |  |  |
| **Title:** | Relationship Between the Eating Assessment Tool-10 and Objective Clinical Ratings of Swallowing Function in Individuals with Head and Neck Cancer | | |  |
|  |  |  |  |  |
| **Author:** | Arrese | **Year:** | 2016 |  |
| **#** | **Question** | | **Outcome** |  |
| **1** | Were the criteria for inclusion in the sample clearly defined? | | Yes |  |
|  |  |  |  |  |
| **2** | Were the study subjects and the setting described in detail? | | Yes |  |
|  |  |  |  |  |
| **3** | Was the exposure measured in a valid and reliable way? | | Yes |  |
|  |  |  |  |  |
| **4** | Were objective, standard criteria used for measurement of the condition? | | Yes |  |
|  |  |  |  |  |
| **5** | Were confounding factors identified? | | Yes |  |
|  |  |  |  |  |
| **6** | Were strategies to deal with confounding factors stated? | | Yes |  |
|  |  |  |  |  |
| **7** | Were the outcomes measured in a valid and reliable way? | | Yes |  |
|  |  |  |  |  |
| **8** | Was appropriate statistical analysis used? | | Yes |  |
|  |  |  |  |  |

| **Lista de chequeo JBI de evaluación crítica para estudios de corte transversal** | | | |  |
| --- | --- | --- | --- | --- |
|  |  |  |  |  |
| **Title:** | Reliability and Validity of the Turkish Eating Assessment Tool (T-EAT-10) | | |  |
|  |  |  |  |  |
| **Author:** | Demir | **Year:** | 2016 |  |
| **#** | **Question** | | **Outcome** |  |
| **1** | Were the criteria for inclusion in the sample clearly defined? | | Yes |  |
|  |  |  |  |  |
| **2** | Were the study subjects and the setting described in detail? | | Yes |  |
|  |  |  |  |  |
| **3** | Was the exposure measured in a valid and reliable way? | | Yes |  |
|  |  |  |  |  |
| **4** | Were objective, standard criteria used for measurement of the condition? | | Yes |  |
|  |  |  |  |  |
| **5** | Were confounding factors identified? | | No |  |
|  |  |  |  |  |
| **6** | Were strategies to deal with confounding factors stated? | | No |  |
|  |  |  |  |  |
| **7** | Were the outcomes measured in a valid and reliable way? | | Yes |  |
|  |  |  |  |  |
| **8** | Was appropriate statistical analysis used? | | Yes |  |
|  |  |  |  |  |

| **JBI Critical Appraisal Checklist for Cross-sectional Studies** | | | |  |
| --- | --- | --- | --- | --- |
|  |  |  |  |  |
| **Title:** | Validation and Cultural Adaptation of the Arabic Version of the Eating Assessment Tool (EAT-10) | | |  |
|  |  |  |  |  |
| **Author:** | Farahat | **Year:** | 2016 |  |
| **#** | **Question** | | **Outcome** |  |
| **1** | Were the criteria for inclusion in the sample clearly defined? | | No |  |
|  |  |  |  |  |
| **2** | Were the study subjects and the setting described in detail? | | No |  |
|  |  |  |  |  |
| **3** | Was the exposure measured in a valid and reliable way? | | Yes |  |
|  |  |  |  |  |
| **4** | Were objective, standard criteria used for measurement of the condition? | | Yes |  |
|  |  |  |  |  |
| **5** | Were confounding factors identified? | | No |  |
|  |  |  |  |  |
| **6** | Were strategies to deal with confounding factors stated? | | No |  |
|  |  |  |  |  |
| **7** | Were the outcomes measured in a valid and reliable way? | | Yes |  |
|  |  |  |  |  |
| **8** | Was appropriate statistical analysis used? | | Yes |  |
|  |  |  |  |  |

| **JBI Critical Appraisal Checklist for Cross-sectional Studies** | | | |  |
| --- | --- | --- | --- | --- |
|  |  |  |  |  |
| **Title:** | Tradução Transcultural e Adaptação do “Eating Assessment Tool” para a Língua Portuguesa em Angola | | |  |
|  |  |  |  |  |
| **Author:** | Leal | **Year:** | 2016 |  |
| **#** | **Question** | | **Outcome** |  |
| **1** | Were the criteria for inclusion in the sample clearly defined? | | Yes |  |
|  |  |  |  |  |
| **2** | Were the study subjects and the setting described in detail? | | Yes |  |
|  |  |  |  |  |
| **3** | Was the exposure measured in a valid and reliable way? | | Yes |  |
|  |  |  |  |  |
| **4** | Were objective, standard criteria used for measurement of the condition? | | Yes |  |
|  |  |  |  |  |
| **5** | Were confounding factors identified? | | No |  |
|  |  |  |  |  |
| **6** | Were strategies to deal with confounding factors stated? | | No |  |
|  |  |  |  |  |
| **7** | Were the outcomes measured in a valid and reliable way? | | Yes |  |
|  |  |  |  |  |
| **8** | Was appropriate statistical analysis used? | | No |  |
|  |  |  |  |  |

| **JBI Critical Appraisal Checklist for Cross-sectional Studies** | | | |  |
| --- | --- | --- | --- | --- |
|  |  |  |  |  |
| **Title:** | Validity and Reliability of the Turkish Version of the Questionnaire for the Assessment of Dysphagia in Multiple Sclerosis | | |  |
|  |  |  |  |  |
| **Author:** | Tenekeci | **Year:** | 2016 |  |
| **#** | **Question** | | **Outcome** |  |
| **1** | Were the criteria for inclusion in the sample clearly defined? | | Yes |  |
|  |  |  |  |  |
| **2** | Were the study subjects and the setting described in detail? | | Yes |  |
|  |  |  |  |  |
| **3** | Was the exposure measured in a valid and reliable way? | | Yes |  |
|  |  |  |  |  |
| **4** | Were objective, standard criteria used for measurement of the condition? | | Yes |  |
|  |  |  |  |  |
| **5** | Were confounding factors identified? | | Yes |  |
|  |  |  |  |  |
| **6** | Were strategies to deal with confounding factors stated? | | Yes |  |
|  |  |  |  |  |
| **7** | Were the outcomes measured in a valid and reliable way? | | Yes |  |
|  |  |  |  |  |
| **8** | Was appropriate statistical analysis used? | | Yes |  |
|  |  |  |  |  |

| **JBI Critical Appraisal Checklist for Cross-sectional Studies** | | | |  |
| --- | --- | --- | --- | --- |
|  |  |  |  |  |
| **Title:** | Reliability and validity of the Chinese Eating Assessment Tool (EAT-10) in evaluation of acute stroke patients with dysphagia | | |  |
|  |  |  |  |  |
| **Author:** | Wong | **Year:** | 2015 |  |
| **#** | **Question** | | **Outcome** |  |
| **1** | Were the criteria for inclusion in the sample clearly defined? | | Yes |  |
|  |  |  |  |  |
| **2** | Were the study subjects and the setting described in detail? | | Yes |  |
|  |  |  |  |  |
| **3** | Was the exposure measured in a valid and reliable way? | | Yes |  |
|  |  |  |  |  |
| **4** | Were objective, standard criteria used for measurement of the condition? | | Yes |  |
|  |  |  |  |  |
| **5** | Were confounding factors identified? | | Yes |  |
|  |  |  |  |  |
| **6** | Were strategies to deal with confounding factors stated? | | Yes |  |
|  |  |  |  |  |
| **7** | Were the outcomes measured in a valid and reliable way? | | Yes |  |
|  |  |  |  |  |
| **8** | Was appropriate statistical analysis used? | | Yes |  |
|  |  |  |  |  |

| **JBI Critical Appraisal Checklist for Cross-sectional Studies** | | | |  |
| --- | --- | --- | --- | --- |
|  |  |  |  |  |
| **Title:** | Criterion validity of the self-report dysphagia assessment tool EAT-10 among neurological patients | | |  |
|  |  |  |  |  |
| **Author:** | Mandysova | **Year:** | 2014 |  |
| **#** | **Question** | | **Outcome** |  |
| **1** | Were the criteria for inclusion in the sample clearly defined? | | Yes |  |
|  |  |  |  |  |
| **2** | Were the study subjects and the setting described in detail? | | Yes |  |
|  |  |  |  |  |
| **3** | Was the exposure measured in a valid and reliable way? | | Yes |  |
|  |  |  |  |  |
| **4** | Were objective, standard criteria used for measurement of the condition? | | No |  |
|  |  |  |  |  |
| **5** | Were confounding factors identified? | | Yes |  |
|  |  |  |  |  |
| **6** | Were strategies to deal with confounding factors stated? | | Yes |  |
|  |  |  |  |  |
| **7** | Were the outcomes measured in a valid and reliable way? | | Yes |  |
|  |  |  |  |  |
| **8** | Was appropriate statistical analysis used? | | No |  |
|  |  |  |  |  |

| **JBI Critical Appraisal Checklist for Cross-sectional Studies** | | | |  |
| --- | --- | --- | --- | --- |
|  |  |  |  |  |
| **Title:** | Sensitivity and specificity of the Eating Assessment Tool and the Volume-Viscosity Swallow Test for clinical evaluation of oropharyngeal dysphagia | | |  |
|  |  |  |  |  |
| **Author:** | Rofes | **Year:** | 2014 |  |
| **#** | **Question** | | **Outcome** |  |
| **1** | Were the criteria for inclusion in the sample clearly defined? | | Yes |  |
|  |  |  |  |  |
| **2** | Were the study subjects and the setting described in detail? | | Yes |  |
|  |  |  |  |  |
| **3** | Was the exposure measured in a valid and reliable way? | | Yes |  |
|  |  |  |  |  |
| **4** | Were objective, standard criteria used for measurement of the condition? | | Yes |  |
|  |  |  |  |  |
| **5** | Were confounding factors identified? | | Yes |  |
|  |  |  |  |  |
| **6** | Were strategies to deal with confounding factors stated? | | Yes |  |
|  |  |  |  |  |
| **7** | Were the outcomes measured in a valid and reliable way? | | Yes |  |
|  |  |  |  |  |
| **8** | Was appropriate statistical analysis used? | | Yes |  |
|  |  |  |  |  |

| **JBI Critical Appraisal Checklist for Cross-sectional Studies** | | | |  |
| --- | --- | --- | --- | --- |
|  |  |  |  |  |
| **Title:** | Reliability and validity of the Italian Eating Assessment Tool | | |  |
|  |  |  |  |  |
| **Author:** | Schindler | **Year:** | 2013 |  |
| **#** | **Question** | | **Outcome** |  |
| **1** | Were the criteria for inclusion in the sample clearly defined? | | Yes |  |
|  |  |  |  |  |
| **2** | Were the study subjects and the setting described in detail? | | Yes |  |
|  |  |  |  |  |
| **3** | Was the exposure measured in a valid and reliable way? | | Yes |  |
|  |  |  |  |  |
| **4** | Were objective, standard criteria used for measurement of the condition? | | Yes |  |
|  |  |  |  |  |
| **5** | Were confounding factors identified? | | Yes |  |
|  |  |  |  |  |
| **6** | Were strategies to deal with confounding factors stated? | | Yes |  |
|  |  |  |  |  |
| **7** | Were the outcomes measured in a valid and reliable way? | | Yes |  |
|  |  |  |  |  |
| **8** | Was appropriate statistical analysis used? | | Yes |  |
|  |  |  |  |  |

| **JBI Critical Appraisal Checklist for Cross-sectional Studies** | | | |  |
| --- | --- | --- | --- | --- |
|  |  |  |  |  |
| **Title:** | Translation and validation of the spanish version of the eating assessment tool-10 (EAT-10) for the screening of dysphagia | | |  |
|  |  |  |  |  |
| **Author:** | Burgos | **Year:** | 2012 |  |
| **#** | **Question** | | **Outcome** |  |
| **1** | Were the criteria for inclusion in the sample clearly defined? | | Yes |  |
|  |  |  |  |  |
| **2** | Were the study subjects and the setting described in detail? | | Yes |  |
|  |  |  |  |  |
| **3** | Was the exposure measured in a valid and reliable way? | | Yes |  |
|  |  |  |  |  |
| **4** | Were objective, standard criteria used for measurement of the condition? | | Yes |  |
|  |  |  |  |  |
| **5** | Were confounding factors identified? | | Yes |  |
|  |  |  |  |  |
| **6** | Were strategies to deal with confounding factors stated? | | Yes |  |
|  |  |  |  |  |
| **7** | Were the outcomes measured in a valid and reliable way? | | Yes |  |
|  |  |  |  |  |
| **8** | Was appropriate statistical analysis used? | | Yes |  |
|  |  |  |  |  |

| **JBI Critical Appraisal Checklist for Cross-sectional Studies** | | | |  |
| --- | --- | --- | --- | --- |
|  |  |  |  |  |
| **Title:** | Cross-cultural adaptation of the Brazilian version of the  Eating Assessment Tool – EAT-10 | | |  |
|  |  |  |  |  |
| **Author:** | Revelo | **Year:** | 2012 |  |
| **#** | **Question** | | **Outcome** |  |
| **1** | Were the criteria for inclusion in the sample clearly defined? | | No |  |
|  |  |  |  |  |
| **2** | Were the study subjects and the setting described in detail? | | No |  |
|  |  |  |  |  |
| **3** | Was the exposure measured in a valid and reliable way? | | Yes |  |
|  |  |  |  |  |
| **4** | Were objective, standard criteria used for measurement of the condition? | | Yes |  |
|  |  |  |  |  |
| **5** | Were confounding factors identified? | | No |  |
|  |  |  |  |  |
| **6** | Were strategies to deal with confounding factors stated? | | Not applicable |  |
|  |  |  |  |  |
| **7** | Were the outcomes measured in a valid and reliable way? | | Yes |  |
|  |  |  |  |  |
| **8** | Was appropriate statistical analysis used? | | Yes |  |
|  |  |  |  |  |

## Cohort Studies

| **JBI Critical Appraisal Checklist for Cohort Studies** | | | |  |
| --- | --- | --- | --- | --- |
|  |  |  |  |  |
| **Title:** | Is EAT-10 Useful to Assess Swallowing during the Chemo-Radiotherapy Phase in Patients with Head and Neck Cancer? A Pilot Study | | |  |
|  |  |  |  |  |
| **Author:** | Bofill-Soler | **Year:** | 2021 |  |
| **#** | **Question** | | **Outcome** |  |
| **1** | Were the two groups similar and recruited from the same population? | | Yes |  |
|  |  |  |  |  |
| **2** | Were the exposures measured similarly to assign people to both exposed and unexposed groups? | | Not applicable |  |
|  |  |  |  |  |
| **3** | Was the exposure measured in a valid and reliable way? | | Yes |  |
|  |  |  |  |  |
| **4** | Were confounding factors identified? | | Not applicable |  |
|  |  |  |  |  |
| **5** | Were strategies to deal with confounding factors stated? | | Not applicable |  |
|  |  |  |  |  |
| **6** | Were the groups/participants free of the outcome at the start of the study (or at the moment of exposure)? | | Not applicable |  |
|  |  |  |  |  |
| **7** | Were the outcomes measured in a valid and reliable way? | | Yes |  |
|  |  |  |  |  |
| **8** | Was the follow up time reported and sufficient to be long enough for outcomes to occur? | | Yes |  |
|  |  |  |  |  |
| **9** | Was follow up complete, and if not, were the reasons to loss to follow up described and explored? | | Yes |  |
|  |  |  |  |  |
| **10** | Were strategies to address incomplete follow up utilized? | | Yes |  |
|  |  |  |  |  |
| **11** | Was appropriate statistical analysis used? | | Yes |  |
|  |  |  |  |  |

| **JBI Critical Appraisal Checklist for Cohort Studies** | | | |  |
| --- | --- | --- | --- | --- |
|  |  |  |  |  |
| **Title:** | Dysphagia symptoms in obstructive sleep apnea: prevalence and clinical correlates | | |  |
|  |  |  |  |  |
| **Author:** | Pizzorni | **Year:** | 2021 |  |
| **#** | **Question** | | **Outcome** |  |
| **1** | Were the two groups similar and recruited from the same population? | | Yes |  |
|  |  |  |  |  |
| **2** | Were the exposures measured similarly to assign people to both exposed and unexposed groups? | | Yes |  |
|  |  |  |  |  |
| **3** | Was the exposure measured in a valid and reliable way? | | Yes |  |
|  |  |  |  |  |
| **4** | Were confounding factors identified? | | Yes |  |
|  |  |  |  |  |
| **5** | Were strategies to deal with confounding factors stated? | | Yes |  |
|  |  |  |  |  |
| **6** | Were the groups/participants free of the outcome at the start of the study (or at the moment of exposure)? | | Not applicable |  |
|  |  |  |  |  |
| **7** | Were the outcomes measured in a valid and reliable way? | | Yes |  |
|  |  |  |  |  |
| **8** | Was the follow up time reported and sufficient to be long enough for outcomes to occur? | | Yes |  |
|  |  |  |  |  |
| **9** | Was follow up complete, and if not, were the reasons to loss to follow up described and explored? | | Yes |  |
|  |  |  |  |  |
| **10** | Were strategies to address incomplete follow up utilized? | | Yes |  |
|  |  |  |  |  |
| **11** | Was appropriate statistical analysis used? | | Yes |  |
|  |  |  |  |  |

| **JBI Critical Appraisal Checklist for Cohort Studies** | | | |  |
| --- | --- | --- | --- | --- |
|  |  |  |  |  |
| **Title:** | Association of positive screening for dysphagia with nutritional status and long-term mortality in hospitalized elderly patients | | |  |
|  |  |  |  |  |
| **Author:** | Mañas-Martínez | **Year:** | 2018 |  |
| **#** | **Question** | | **Outcome** |  |
| **1** | Were the two groups similar and recruited from the same population? | | Yes |  |
|  |  |  |  |  |
| **2** | Were the exposures measured similarly to assign people to both exposed and unexposed groups? | | Yes |  |
|  |  |  |  |  |
| **3** | Was the exposure measured in a valid and reliable way? | | Yes |  |
|  |  |  |  |  |
| **4** | Were confounding factors identified? | | Yes |  |
|  |  |  |  |  |
| **5** | Were strategies to deal with confounding factors stated? | | Yes |  |
|  |  |  |  |  |
| **6** | Were the groups/participants free of the outcome at the start of the study (or at the moment of exposure)? | | Not applicable |  |
|  |  |  |  |  |
| **7** | Were the outcomes measured in a valid and reliable way? | | Yes |  |
|  |  |  |  |  |
| **8** | Was the follow up time reported and sufficient to be long enough for outcomes to occur? | | Yes |  |
|  |  |  |  |  |
| **9** | Was follow up complete, and if not, were the reasons to loss to follow up described and explored? | | Yes |  |
|  |  |  |  |  |
| **10** | Were strategies to address incomplete follow up utilized? | | Yes |  |
|  |  |  |  |  |
| **11** | Was appropriate statistical analysis used? | | Yes |  |
|  |  |  |  |  |

| **JBI Critical Appraisal Checklist for Cohort Studies** | | | |  |
| --- | --- | --- | --- | --- |
|  |  |  |  |  |
| **Title:** | Reliability and validity of the Eating Assessment Tool-10 (Greek adaptation) in neurogenic and head and neck cancer-related oropharyngeal dysphagia | | |  |
|  |  |  |  |  |
| **Author:** | Printza | **Year:** | 2018 |  |
| **#** | **Question** | | **Outcome** |  |
| **1** | Were the two groups similar and recruited from the same population? | | Yes |  |
|  |  |  |  |  |
| **2** | Were the exposures measured similarly to assign people to both exposed and unexposed groups? | | Yes |  |
|  |  |  |  |  |
| **3** | Was the exposure measured in a valid and reliable way? | | Yes |  |
|  |  |  |  |  |
| **4** | Were confounding factors identified? | | Yes |  |
|  |  |  |  |  |
| **5** | Were strategies to deal with confounding factors stated? | | Yes |  |
|  |  |  |  |  |
| **6** | Were the groups/participants free of the outcome at the start of the study (or at the moment of exposure)? | | Not applicable |  |
|  |  |  |  |  |
| **7** | Were the outcomes measured in a valid and reliable way? | | Yes |  |
|  |  |  |  |  |
| **8** | Was the follow up time reported and sufficient to be long enough for outcomes to occur? | | Not applicable |  |
|  |  |  |  |  |
| **9** | Was follow up complete, and if not, were the reasons to loss to follow up described and explored? | | Not applicable |  |
|  |  |  |  |  |
| **10** | Were strategies to address incomplete follow up utilized? | | Not applicable |  |
|  |  |  |  |  |
| **11** | Was appropriate statistical analysis used? | | Yes |  |
|  |  |  |  |  |

| **JBI Critical Appraisal Checklist for Cohort Studies** | | | |  |
| --- | --- | --- | --- | --- |
|  |  |  |  |  |
| **Title:** | Validation of the Spanish Version of the Eating Assessment Tool-10 (EAT-10spa) in Colombia. A Blinded Prospective Cohort Study | | |  |
|  |  |  |  |  |
| **Author:** | Giraldo-Cadavid | **Year:** | 2016 |  |
| **#** | **Question** | | **Outcome** |  |
| **1** | Were the two groups similar and recruited from the same population? | | Yes |  |
|  |  |  |  |  |
| **2** | Were the exposures measured similarly to assign people to both exposed and unexposed groups? | | Yes |  |
|  |  |  |  |  |
| **3** | Was the exposure measured in a valid and reliable way? | | Yes |  |
|  |  |  |  |  |
| **4** | Were confounding factors identified? | | Yes |  |
|  |  |  |  |  |
| **5** | Were strategies to deal with confounding factors stated? | | Yes |  |
|  |  |  |  |  |
| **6** | Were the groups/participants free of the outcome at the start of the study (or at the moment of exposure)? | | Not applicable |  |
|  |  |  |  |  |
| **7** | Were the outcomes measured in a valid and reliable way? | | Yes |  |
|  |  |  |  |  |
| **8** | Was the follow up time reported and sufficient to be long enough for outcomes to occur? | | Yes |  |
|  |  |  |  |  |
| **9** | Was follow up complete, and if not, were the reasons to loss to follow up described and explored? | | Yes |  |
|  |  |  |  |  |
| **10** | Were strategies to address incomplete follow up utilized? | | Yes |  |
|  |  |  |  |  |
| **11** | Was appropriate statistical analysis used? | | Yes |  |
|  |  |  |  |  |

| **JBI Critical Appraisal Checklist for Cohort Studies** | | | |  |
| --- | --- | --- | --- | --- |
|  |  |  |  |  |
| **Title:** | Validation of the Swedish translation of eating assessment tool (S-EAT-10) | | |  |
|  |  |  |  |  |
| **Author:** | Moller | **Year:** | 2016 |  |
| **#** | **Question** | | **Outcome** |  |
| **1** | Were the two groups similar and recruited from the same population? | | Yes |  |
|  |  |  |  |  |
| **2** | Were the exposures measured similarly to assign people to both exposed and unexposed groups? | | Yes |  |
|  |  |  |  |  |
| **3** | Was the exposure measured in a valid and reliable way? | | Yes |  |
|  |  |  |  |  |
| **4** | Were confounding factors identified? | | Yes |  |
|  |  |  |  |  |
| **5** | Were strategies to deal with confounding factors stated? | | Yes |  |
|  |  |  |  |  |
| **6** | Were the groups/participants free of the outcome at the start of the study (or at the moment of exposure)? | | Not applicable |  |
|  |  |  |  |  |
| **7** | Were the outcomes measured in a valid and reliable way? | | Yes |  |
|  |  |  |  |  |
| **8** | Was the follow up time reported and sufficient to be long enough for outcomes to occur? | | Yes |  |
|  |  |  |  |  |
| **9** | Was follow up complete, and if not, were the reasons to loss to follow up described and explored? | | Yes |  |
|  |  |  |  |  |
| **10** | Were strategies to address incomplete follow up utilized? | | Yes |  |
|  |  |  |  |  |
| **11** | Was appropriate statistical analysis used? | | Yes |  |
|  |  |  |  |  |

| **JBI Critical Appraisal Checklist for Cohort Studies** | | | |  |
| --- | --- | --- | --- | --- |
|  |  |  |  |  |
| **Title:** | Measuring Outcomes for Dysphagia: Validity and Reliability of the European Portuguese Eating Assessment Tool (P-EAT-10) | | |  |
|  |  |  |  |  |
| **Author:** | Nogueira | **Year:** | 2015 |  |
| **#** | **Question** | | **Outcome** |  |
| **1** | Were the two groups similar and recruited from the same population? | | Yes |  |
|  |  |  |  |  |
| **2** | Were the exposures measured similarly to assign people to both exposed and unexposed groups? | | Yes |  |
|  |  |  |  |  |
| **3** | Was the exposure measured in a valid and reliable way? | | Yes |  |
|  |  |  |  |  |
| **4** | Were confounding factors identified? | | Yes |  |
|  |  |  |  |  |
| **5** | Were strategies to deal with confounding factors stated? | | Yes |  |
|  |  |  |  |  |
| **6** | Were the groups/participants free of the outcome at the start of the study (or at the moment of exposure)? | | Not applicable |  |
|  |  |  |  |  |
| **7** | Were the outcomes measured in a valid and reliable way? | | Yes |  |
|  |  |  |  |  |
| **8** | Was the follow up time reported and sufficient to be long enough for outcomes to occur? | | Not applicable |  |
|  |  |  |  |  |
| **9** | Was follow up complete, and if not, were the reasons to loss to follow up described and explored? | | Yes |  |
|  |  |  |  |  |
| **10** | Were strategies to address incomplete follow up utilized? | | Yes |  |
|  |  |  |  |  |
| **11** | Was appropriate statistical analysis used? | | Yes |  |
|  |  |  |  |  |

| **JBI Critical Appraisal Checklist for Cohort Studies** | | | |  |
| --- | --- | --- | --- | --- |
|  |  |  |  |  |
| **Title:** | The Ability of the 10-Item Eating Assessment Tool (EAT-10) to Predict Aspiration Risk in Persons With Dysphagia | | |  |
|  |  |  |  |  |
| **Author:** | Cheney | **Year:** | 2014 |  |
| **#** | **Question** | | **Outcome** |  |
| **1** | Were the two groups similar and recruited from the same population? | | Yes |  |
|  |  |  |  |  |
| **2** | Were the exposures measured similarly to assign people to both exposed and unexposed groups? | | Not applicable |  |
|  |  |  |  |  |
| **3** | Was the exposure measured in a valid and reliable way? | | Yes |  |
|  |  |  |  |  |
| **4** | Were confounding factors identified? | | Yes |  |
|  |  |  |  |  |
| **5** | Were strategies to deal with confounding factors stated? | | Yes |  |
|  |  |  |  |  |
| **6** | Were the groups/participants free of the outcome at the start of the study (or at the moment of exposure)? | | Not applicable |  |
|  |  |  |  |  |
| **7** | Were the outcomes measured in a valid and reliable way? | | Yes |  |
|  |  |  |  |  |
| **8** | Was the follow up time reported and sufficient to be long enough for outcomes to occur? | | Not applicable |  |
|  |  |  |  |  |
| **9** | Was follow up complete, and if not, were the reasons to loss to follow up described and explored? | | Not applicable |  |
|  |  |  |  |  |
| **10** | Were strategies to address incomplete follow up utilized? | | Not applicable |  |
|  |  |  |  |  |
| **11** | Was appropriate statistical analysis used? | | Yes |  |
|  |  |  |  |  |

| **JBI Critical Appraisal Checklist for Cohort Studies** | | | |  |
| --- | --- | --- | --- | --- |
|  |  |  |  |  |
| **Title:** | Validity and Reliability of the Eating Assessment Tool (EAT-10) | | |  |
|  |  |  |  |  |
| **Author:** | Belafsky | **Year:** | 2008 |  |
| **#** | **Question** | | **Outcome** |  |
| **1** | Were the two groups similar and recruited from the same population? | | Yes |  |
|  |  |  |  |  |
| **2** | Were the exposures measured similarly to assign people to both exposed and unexposed groups? | | Yes |  |
|  |  |  |  |  |
| **3** | Was the exposure measured in a valid and reliable way? | | Yes |  |
|  |  |  |  |  |
| **4** | Were confounding factors identified? | | Yes |  |
|  |  |  |  |  |
| **5** | Were strategies to deal with confounding factors stated? | | Yes |  |
|  |  |  |  |  |
| **6** | Were the groups/participants free of the outcome at the start of the study (or at the moment of exposure)? | | Not applicable |  |
|  |  |  |  |  |
| **7** | Were the outcomes measured in a valid and reliable way? | | Yes |  |
|  |  |  |  |  |
| **8** | Was the follow up time reported and sufficient to be long enough for outcomes to occur? | | Not applicable |  |
|  |  |  |  |  |
| **9** | Was follow up complete, and if not, were the reasons to loss to follow up described and explored? | | Not applicable |  |
|  |  |  |  |  |
| **10** | Were strategies to address incomplete follow up utilized? | | Not applicable |  |
|  |  |  |  |  |
| **11** | Was appropriate statistical analysis used? | | Not applicable |  |
|  |  |  |  |  |

## Systematic reviews

| AMSTAR 2 assessment for the methodological quality of systematic reviews | | | | | | | | | | | | | | | |  |
| --- | --- | --- | --- | --- | --- | --- | --- | --- | --- | --- | --- | --- | --- | --- | --- | --- |
|  |  |  |  |  |  |  |  |  |  |  |  |  |  |  |  |  |
| **Title:** | Cultural Adaptation and Validation of Questionnaires for Evaluation of Health‑Related Quality of Life with Dysphagia in Different Countries: A Systematic Review | | | | | | | | | | | | | | |  |
|  |  |  |  |  |  |  |  |  |  |  |  |  |  |  |  |  |
| **Autor:** | Yang | | | | | **Year:** | 2021 | | | | | | | | |  |
|  |  |  |  |  |  |  |  |  |  | |  | |  | |  |  |
| 1. Did the research questions and inclusion criteria for the review include the components of PICO? | | | | | | | | | |  | | YES | | **X** | |  |
|  |  |  |  |  |  |  |  |  |  |  | | NO | |  | |  |
| 2. Did the report of the review contain an explicit statement that the review methods were established prior to the conduct of the review and did the report justify any significant deviations from the protocol? | | | | | | | | | |  | | YES | |  | |  |
|  |  |  |  |  |  |  |  |  |  |  | | PARCIAL YES | |  | |  |
|  |  |  |  |  |  |  |  |  |  |  | | NO | | **X** | |  |
| 3. Did the review authors explain their selection of the study designs for inclusion in the review? | | | | | | | | | |  | | YES | | **X** | |  |
|  |  |  |  |  |  |  |  |  |  |  | | NO | |  | |  |
| 4. Did the review authors use a comprehensive literature search strategy? | | | | | | | | | |  | | YES | |  | |  |
|  |  |  |  |  |  |  |  |  |  |  | | PARCIAL YES | | **X** | |  |
|  |  |  |  |  |  |  |  |  |  |  | | NO | |  | |  |
| 5. Did the review authors perform study selection in duplicate? | | | | | | | | | |  | | YES | | **X** | |  |
|  |  |  |  |  |  |  |  |  |  |  | | NO | |  | |  |
| 6. Did the review authors perform data extraction in duplicate? | | | | | | | | | |  | | YES | | **X** | |  |
|  |  |  |  |  |  |  |  |  |  |  | | NO | |  | |  |
| 7. Did the review authors provide a list of excluded studies and justify the exclusions? | | | | | | | | | |  | | YES | |  | |  |
|  |  |  |  |  |  |  |  |  |  |  | | PARCIAL YES | | **X** | |  |
|  |  |  |  |  |  |  |  |  |  |  | | NO | |  | |  |
| 8. Did the review authors describe the included studies in adequate detail? | | | | | | | | | |  | | YES | | **X** | |  |
|  |  |  |  |  |  |  |  |  |  |  | | PARCIAL YES | |  | |  |
|  |  |  |  |  |  |  |  |  |  |  | | NO | |  | |  |
| 9. Did the review authors use a satisfactory technique for assessing the risk of bias (RoB) in individual studies that were included in the review? | | | | | | | | | | RCTs | | YES | |  | |  |
|  |  |  |  |  |  |  |  |  |  |  |  | PARCIAL YES | |  | |  |
|  |  |  |  |  |  |  |  |  |  |  |  | NO | |  | |  |
|  |  |  |  |  |  |  |  |  |  |  |  | Includes only NRSI | |  | |  |
|  |  |  |  |  |  |  |  |  |  | NRSI | | YES | | **X** | |  |
|  |  |  |  |  |  |  |  |  |  |  |  | PARCIAL YES | |  | |  |
|  |  |  |  |  |  |  |  |  |  |  |  | NO | |  | |  |
|  |  |  |  |  |  |  |  |  |  |  |  | Includes only RCTs | |  | |  |
| 10. Did the review authors report on the sources of funding for the studies included in the review? | | | | | | | | | |  | | YES | |  | |  |
|  |  |  |  |  |  |  |  |  |  |  | | NO | | **X** | |  |
| 11. If meta-analysis was performed did the review authors use appropriate methods for statistical combination of results? | | | | | | | | | | RCTs | | YES | |  | |  |
|  |  |  |  |  |  |  |  |  |  |  |  | NO | |  | |  |
|  |  |  |  |  |  |  |  |  |  |  |  | NO META-ANALYSIS CONDUCTED | |  | |  |
|  |  |  |  |  |  |  |  |  |  | NRSI | | YES | |  | |  |
|  |  |  |  |  |  |  |  |  |  |  |  | NO | |  | |  |
|  |  |  |  |  |  |  |  |  |  |  |  | NO META-ANALYSIS CONDUCTED | | **X** | |  |
| 12. If meta-analysis was performed, did the review authors assess the potential impact of RoB in individual studies on the results of the meta-analysis or other evidence synthesis? | | | | | | | | | |  | | YES | |  | |  |
|  |  |  |  |  |  |  |  |  |  |  | | NO | |  | |  |
|  |  |  |  |  |  |  |  |  |  |  | | NO META-ANALYSIS CONDUCTED | | **X** | |  |
| 13. Did the review authors account for RoB in individual studies when interpreting/ discussing the results of the review? | | | | | | | | | |  | | YES | | **X** | |  |
|  |  |  |  |  |  |  |  |  |  |  | | NO | |  | |  |
| 14. Did the review authors provide a satisfactory explanation for, and discussion of, any heterogeneity observed in the results of the review? | | | | | | | | | |  | | YES | | **X** | |  |
|  |  |  |  |  |  |  |  |  |  |  | | NO | |  | |  |
| 15. If they performed quantitative synthesis, did the review authors carry out an adequate investigation of publication bias (small study bias) and discuss its likely impact on the results of the review? | | | | | | | | | |  | | YES | |  | |  |
|  |  |  |  |  |  |  |  |  |  |  | | NO | |  | |  |
|  |  |  |  |  |  |  |  |  |  |  | | NO META-ANALYSIS CONDUCTED | | **X** | |  |
| 16. Did the review authors report any potential sources of conflict of interest, including any funding they received for conducting the review? | | | | | | | | | |  | | YES | | **X** | |  |
|  |  |  |  |  |  |  |  |  |  |  | | NO | |  | |  |
|  |  |  |  |  |  |  |  |  |  | |  | |  | |  |  |
| Overall quality of systematic review: | | | | | | | | | | | | **MEDIUM** | | | |  |

| AMSTAR 2 assessment for the methodological quality of systematic reviews | | | | | | | | | | | | | | | |  |
| --- | --- | --- | --- | --- | --- | --- | --- | --- | --- | --- | --- | --- | --- | --- | --- | --- |
|  |  |  |  |  |  |  |  |  |  |  |  |  |  |  |  |  |
| **Title:** | Psychometric properties of questionnaires on functional health status in oropharyngeal dysphagia: A systematic literature review | | | | | | | | | | | | | | |  |
|  |  |  |  |  |  |  |  |  |  |  |  |  |  |  |  |  |
| **Author:** | Speyer | | | | | **Year:** | 2014 | | | | | | | | |  |
|  |  |  |  |  |  |  |  |  |  | |  | |  | |  |  |
| 1. Did the research questions and inclusion criteria for the review include the components of PICO? | | | | | | | | | |  | | YES | | **X** | |  |
|  |  |  |  |  |  |  |  |  |  |  | | NO | |  | |  |
| 2. Did the report of the review contain an explicit statement that the review methods were established prior to the conduct of the review and did the report justify any significant deviations from the protocol? | | | | | | | | | |  | | YES | |  | |  |
|  |  |  |  |  |  |  |  |  |  |  | | PARCIAL YES | |  | |  |
|  |  |  |  |  |  |  |  |  |  |  | | NO | | **X** | |  |
| 3. Did the review authors explain their selection of the study designs for inclusion in the review? | | | | | | | | | |  | | YES | | **X** | |  |
|  |  |  |  |  |  |  |  |  |  |  | | NO | |  | |  |
| 4. Did the review authors use a comprehensive literature search strategy? | | | | | | | | | |  | | YES | |  | |  |
|  |  |  |  |  |  |  |  |  |  |  | | PARCIAL YES | | **X** | |  |
|  |  |  |  |  |  |  |  |  |  |  | | NO | |  | |  |
| 5. Did the review authors perform study selection in duplicate? | | | | | | | | | |  | | YES | | **X** | |  |
|  |  |  |  |  |  |  |  |  |  |  | | NO | |  | |  |
| 6. Did the review authors perform data extraction in duplicate? | | | | | | | | | |  | | YES | | **X** | |  |
|  |  |  |  |  |  |  |  |  |  |  | | NO | |  | |  |
| 7. Did the review authors provide a list of excluded studies and justify the exclusions? | | | | | | | | | |  | | YES | |  | |  |
|  |  |  |  |  |  |  |  |  |  |  | | PARCIAL YES | | **X** | |  |
|  |  |  |  |  |  |  |  |  |  |  | | NO | |  | |  |
| 8. Did the review authors describe the included studies in adequate detail? | | | | | | | | | |  | | YES | | **X** | |  |
|  |  |  |  |  |  |  |  |  |  |  | | PARCIAL YES | |  | |  |
|  |  |  |  |  |  |  |  |  |  |  | | NO | |  | |  |
| 9. Did the review authors use a satisfactory technique for assessing the risk of bias (RoB) in individual studies that were included in the review? | | | | | | | | | | RCTs | | YES | |  | |  |
|  |  |  |  |  |  |  |  |  |  |  |  | PARCIAL YES | |  | |  |
|  |  |  |  |  |  |  |  |  |  |  |  | NO | |  | |  |
|  |  |  |  |  |  |  |  |  |  |  |  | Includes only NRSI | |  | |  |
|  |  |  |  |  |  |  |  |  |  | NRSI | | YES | | **X** | |  |
|  |  |  |  |  |  |  |  |  |  |  |  | PARCIAL YES | |  | |  |
|  |  |  |  |  |  |  |  |  |  |  |  | NO | |  | |  |
|  |  |  |  |  |  |  |  |  |  |  |  | Includes only RCTs | |  | |  |
| 10. Did the review authors report on the sources of funding for the studies included in the review? | | | | | | | | | |  | | YES | |  | |  |
|  |  |  |  |  |  |  |  |  |  |  | | NO | | **X** | |  |
| 11. If meta-analysis was performed did the review authors use appropriate methods for statistical combination of results? | | | | | | | | | | RCTs | | YES | |  | |  |
|  |  |  |  |  |  |  |  |  |  |  |  | NO | |  | |  |
|  |  |  |  |  |  |  |  |  |  |  |  | NO META-ANALYSIS CONDUCTED | |  | |  |
|  |  |  |  |  |  |  |  |  |  | NRSI | | YES | |  | |  |
|  |  |  |  |  |  |  |  |  |  |  |  | NO | |  | |  |
|  |  |  |  |  |  |  |  |  |  |  |  | NO META-ANALYSIS CONDUCTED | | **X** | |  |
| 12. If meta-analysis was performed, did the review authors assess the potential impact of RoB in individual studies on the results of the meta-analysis or other evidence synthesis? | | | | | | | | | |  | | YES | |  | |  |
|  |  |  |  |  |  |  |  |  |  |  | | NO | |  | |  |
|  |  |  |  |  |  |  |  |  |  |  | | NO META-ANALYSIS CONDUCTED | | **X** | |  |
| 13. Did the review authors account for RoB in individual studies when interpreting/ discussing the results of the review? | | | | | | | | | |  | | YES | | **X** | |  |
|  |  |  |  |  |  |  |  |  |  |  | | NO | |  | |  |
| 14. Did the review authors provide a satisfactory explanation for, and discussion of, any heterogeneity observed in the results of the review? | | | | | | | | | |  | | YES | | **X** | |  |
|  |  |  |  |  |  |  |  |  |  |  | | NO | |  | |  |
| 15. If they performed quantitative synthesis, did the review authors carry out an adequate investigation of publication bias (small study bias) and discuss its likely impact on the results of the review? | | | | | | | | | |  | | YES | |  | |  |
|  |  |  |  |  |  |  |  |  |  |  | | NO | |  | |  |
|  |  |  |  |  |  |  |  |  |  |  | | NO META-ANALYSIS CONDUCTED | | **X** | |  |
| 16. Did the review authors report any potential sources of conflict of interest, including any funding they received for conducting the review? | | | | | | | | | |  | | YES | | **X** | |  |
|  |  |  |  |  |  |  |  |  |  |  | | NO | |  | |  |
|  |  |  |  |  |  |  |  |  |  | |  | |  | |  |  |
| Overall quality of systematic review: | | | | | | | | | | | | **MEDIUM** | | | |  |
